# MAP65 Coordinate Microtubule Growth during Bundle Formation

**DOI:** 10.1371/journal.pone.0056808

**Published:** 2013-02-21

**Authors:** Virginie Stoppin-Mellet, Vincent Fache, Didier Portran, Jean-Louis Martiel, Marylin Vantard

**Affiliations:** Laboratoaire de Physiologie Cellulaire & Végétale, Institut de Recherches en Technologies et Sciences pour le Vivant (iRTSV), Centre National de la Recherche Scientifique/Commissariat à l’énergie atomique et aux énergies alternatives/Institut National de la Recherche Agronomique/Université Joseph Fourier (CNRS/CEA/INRA/UJF), Grenoble, France; Cancer Research UK London Research Institute, United Kingdom

## Abstract

Microtubules (MTs) are highly dynamical structures that play a crucial role in cell physiology. In cooperation with microtubule-associated proteins (MAPs), MTs form bundles endowing cells with specific mechanisms to control their shape or generate forces. Whether the dynamics of MTs is affected by the lateral connections that MAPs make between MTs during bundle formation is still under debate. Using *in vitro* reconstitution of MT bundling, we analyzed the dynamics of MT bundles generated by two plant MAP65 (MAP65-1/4), MAP65-1 being the plant ortholog of vertebrate PRC1 and yeast Ase1. MAP65-1/4 limit the amplitude of MT bundle depolymerization and increase the elongation phases. The subsequent sustained elongation of bundles is governed by the coordination of MT growth, so that MT ends come in close vicinity. We develop a model based on the assumption that both MAP65-1/4 block MT depolymerization. Model simulations reveal that rescue frequencies are higher between parallel than between anti-parallel MTs. In consequence the polarity of bundled MTs by MAP65 controls the amplitude of bundle’s growth. Our results illustrate how MAP-induced MT-bundling, which is finely tuned by MT polarity, robustly coordinates MT elongation within bundles.

## Introduction

Microtubules (MTs) are dynamic polar polymers assembled from tubulin heterodimers and alternating polymerization and depolymerization phases at their ends [1]. MT catastrophes (switch from growth to shrinkage) or rescues (reverse switch from pause or shrinkage to growth) events have received much attention *in vivo* [2], [3], *in vitro* [4] and in theoretical studies [5]. These sudden transitions of MT dynamics, named dynamic instability, coordinate many aspects of cellular processes crucial to the establishment of cell polarity, cell morphogenesis or cell division. Powering these cellular functions often hinges on the ability of MTs to self-organize into highly ordered structures. This is true for most differentiated animal cell types that contain large numbers of MT arrays in non-centrosomal assembly (e.g. neuronal cells, polarized epithelial cells), in most fungi and in acentrosomal plant cells [6]. In these eukaryote cells, many MT arrays are composed of bundles. Although these MTs are cross-linked, they stay dynamic inside bundles [7]. How the dynamic behavior of the MTs and their polarity within the bundles affect the steady-state of these MT networks and their self-organization into highly ordered arrays remains an important question in cell biology.

MT bundles are built through the activity of cross-linking proteins, including motor and/or non-motor microtubule-associated proteins (MAPs), among them the members of the non-motor MAP65 family. These MAP65 are major MT cross-linkers in vertebrate (PRC1, [8]), fungi (Ase1, [9]) and plant cells (MAP65-1 to MAP65-9, [10]). *In vivo*, MAP65 proteins are required for the maintenance of the mitotic spindle bipolarity during midzone formation in anaphase and for cytokinesis [11], [12], [13], [14]. Ase1 also mediates the bundling of interphase MTs [13]. In plant, with the exception of MAP65-3, direct evidence for specific roles of the different MAP65 in MT bundling *in vivo* are still missing. Nevertheless, all plant MAP65 studied so far are associated with MT bundles *in vivo*, but their cellular localizations vary. For example, MAP65-1 associates with cortical MTs, the pre-prophase band and the spindle mid-zone [15], [16], whereas MAP65-4 is associated with the forming spindle (prophase) and the kinetochore fibers (prophase to anaphase; [17]). *In vitro*, all these MAP65 share the common ability to bundle MTs, but they differ by their physical interactions with the MT lattice and/or by the cross-links they form between adjacent MTs. Indeed, *in vitro,* MAP65-1 and PRC1 cross-link anti-parallel MTs with an inter-MT spacing of 30 nm. In addition, the cross-links between MTs are diagonally oriented with an angle of ∼60° relative to the MT lattice [18], [19]. In marked contrast, MAP65-4 links both parallel and anti-parallel MTs by forming perpendicular cross-bridges of 15 nm wide [17]. Ase1 forms cross-links of 6 nm mostly between anti-parallel MTs [9], [20] but the structure of the cross-bridges between MTs has not yet been determined. Thus the specific physico-chemical properties of MAP65-1/4 isovariants might allow the formation of bundles with different *in vitro* dynamics that would explain their specific distribution and function *in vivo*.

Up to now, little is known about the dynamic of MTs inside bundles. One on the reason for this lack of data is that the analysis of the dynamics of bundled MTs in living cells is difficult due to the high MT density within bundles such as in the plant cell cortex or in the mitotic spindle. In consequence, the tracking of MT dynamics inside a bundle is often difficult. For example, the effects of neuronal MT bundlers tau and MAP2 on dynamics of cross-linked MTs are poorly known, although they have been extensively studied on single MTs [21]. As well, several studies showed that PRC1, Ase1 and MAP65-4 do not affect MT growth and shrinkage velocities of bundled MTs [13], [17], [20], [22]. Furthermore, bundled MTs associated with MAP65-1 in plant living cells have comparable growth and shortening velocities than unbundled MTs [23]. Whether other parameters of MT dynamics are modulated upon bundling remains to be investigated. Although, some experimental evidences suggest that MAP65-4 limits catastrophe events of bundled MTs *in vitro* [17]. Finally, it is not known if the global dynamic of MT bundles generated by various MAPs results from intrinsic MT polymerization activity and/or from MT-MT coupling inside the bundle. In particular, whether MAP-driven MT orientation (parallel versus anti-parallel orientation) and MT organization (distance between adjacent MTs, stability of the cross-links) within bundles affect MT bundle growth is still unclear.

In the present study, we asked whether MT bundling affects the dynamics of MT inside bundles, and whether MT orientation controls the growth of the whole bundle. To address these questions, we focused on MAP65-1 and MAP65-4 as both proteins differ by their selectivity for parallel and anti-parallel MTs and by the geometry of the inter-MT links they form. We set up a biomimetic assay that reproduces the assembly of bundles made of numerous MTs to mimic *in vivo* MT bundles. We found that both MAP65-1/4 drive bundled MT dynamics into an unlimited growth regime by reducing MT catastrophe and increasing MT rescue events without affecting MT growth and shortening rates. We then developed a model to correlate the MT polarity and the dynamic properties of individual MTs in MAP65 induced bundles with the observed bundle growth *in vitro*. Our experimental data and simulations show that bundled MTs adopt a collective behavior resulting from coordinated MT growth. In addition, we found that the polarity of MTs linked by MAP65 finely tunes the growth rate of bundles.

## Materials and Methods

### Preparation of Proteins

Tubulin was purified from bovine brain according to [24] and assembled in BRB buffer (80 mM PIPES, pH 6.8, 1 mM EGTA, and 1 mM MgCl_2_ plus 1 mM GTP). Bovine brains were obtained from a commercial slaughterhouse in Grenoble. Fluorescent tubulin (Alexa 488-labelled tubulin and Alexa 568-labelled tubulin) and biotinylated tubulin were prepared according to [25]. Recombinant *Arabidopsis thaliana* MAP65-1 and MAP65-4 were purified as tagged proteins with a His tag at the N and C termini, or GFP at the N terminus and His tag at the C terminus as described by [18]. They were purified on Ni Sepharose columns and stored at −80°C in 10% (v/v) glycerol, 50 mM NaPi, 0.1 M NaCl, and 0.5 mM DTT, pH 7.9.

### Total Internal Reflection Fluorescence Microscopy Assays

**Figure 1:**
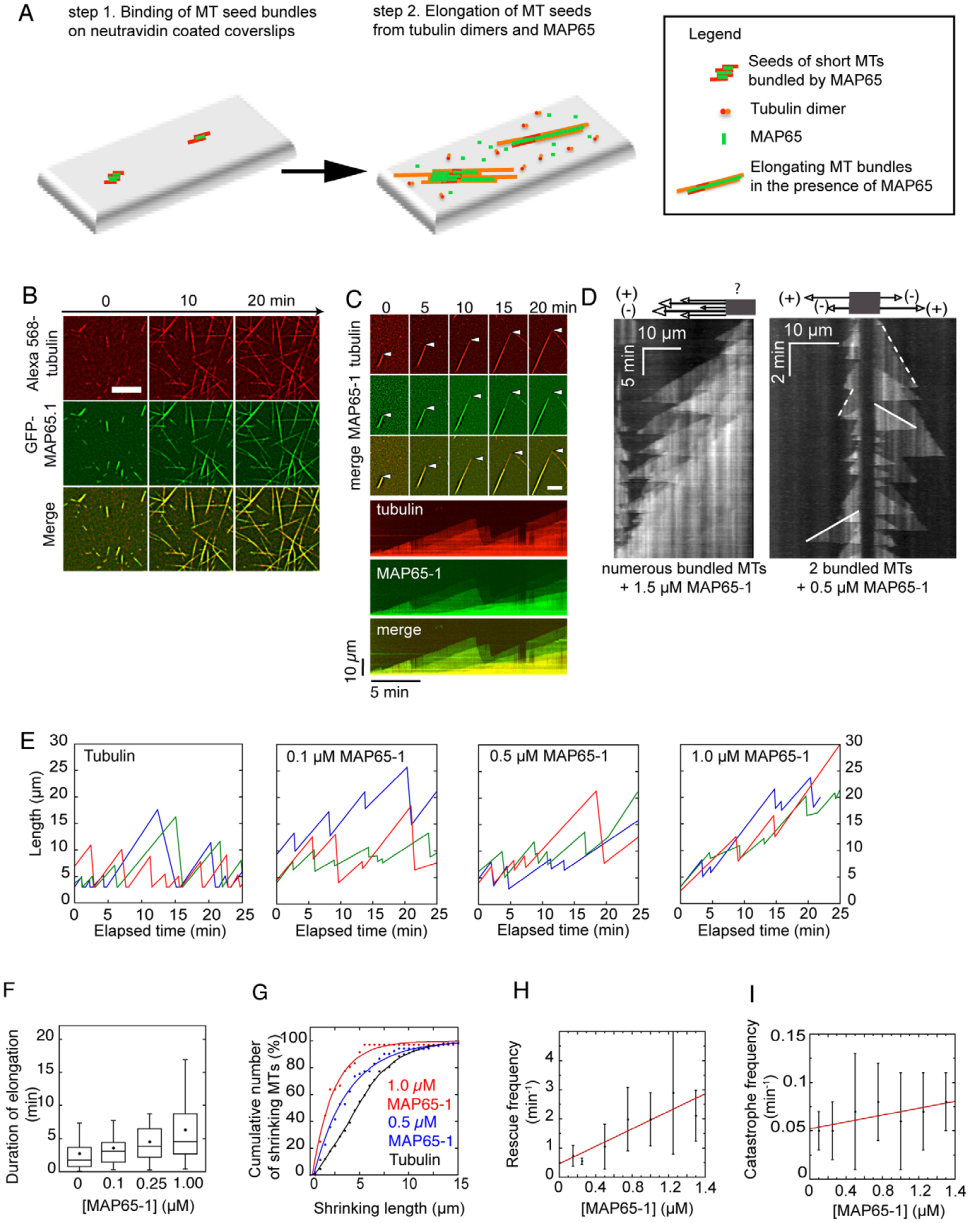
Dynamics of MAP65-1 bundled MTs. (**A**)**.** Scheme of the experimental set-up. MT seed bundles consist of short stable fluorescent and biotinylated MTs cross-linked by MAP65. These MT seed bundles are attached to neutravidin-coated coverslips (step 1). Dynamic MTs elongate from the bundle seeds in the presence of fluorescent tubulin and GFP-MAP65 (step 2). (**B**)**.** Time-lapse of bundles elongating from MT seed bundles in the presence of GFP-MAP65-1 (0.5 µM) observed by TIRF microscopy. Scale bar, 10 µm (**C**). Top panel: Time-lapse of an elongating bundle (arrowhead) simultaneously observed in the red Alexa 568-tubulin channel (top row) and green GFP-MAP65-1 channel (middle row). Lower row is the merged image. Bottom panel: Kymographs of the elongating bundle highlighted by the arrowhead. Tubulin (red) and GFP-MAP65-1 (green) traces are superimposable, indicating the binding of MAP65-1 is concomitant with MT elongation. (**D**)**.** Examples of kymographs of MT bundles. Solid lines correspond to MT plus ends that elongate rapidly, and dotted lines correspond to MT minus ends. Schemes on top of the kymographs show the putative organization of MTs in the bundle. The kymograph shown on the left corresponds to a bundle with a large but unknown number of MTs. The right kymograph shows a bundle with two anti-parallel MTs. (**E**)**.** Length history plots of 3 single MTs in the absence of MAP65-1 (left panel), and 3 MT bundles in the presence of increasing concentrations of MAP65-1 (three right panels). (**F**)**.** Duration of elongation phases after a rescue event as a function of MAP65-1 concentration. Black dots indicate the mean values. (**G**)**.** Depolymerization length of MTs in the presence of various concentrations of MAP65-1. (**H–I**)**.** Estimation of rescue frequencies (F) and catastrophe frequencies (G) of MTs in bundles as a function of MAP65-1 concentration. The slope of the linear fit for catastrophe frequencies is not significantly different from 0.

Assays were done according to [17]. Briefly, assays were performed either in 5 µl final volume between a slide and a coverslip, or in a perfusion chamber (15 µl). Glass was cleaned with rounds of ethanol/water washes, and further silanized using dichloromethylsilan to limit interactions between MTs and the cover glass. GMPCPP-stabilized short MTs were assembled from 10 µM tubulin (3 µM Alexa 568-labelled tubulin, 7 µM biotinylated tubulin) with 0.5 mM GMPCPP in BRB buffer, for 2 hours at 37°C. These short MTs (0.3 µM) were incubated with MAP65-1 (0.1 µM MAP65-1/4) for 10 min at room temperature to generate MT seed bundles. They were then laid on neutravidin-coated slides for 10 min. After two washes with BRB-1%BSA, MT seed bundles were incubated with an elongation mix containing tubulin (17 µM of unlabeled tubulin, 5 µM of Alexa-labeled tubulin), MAP65-1/4 (from 0.1 µM to 1.5 µM), 1 mM GTP, an anti-fading cocktail (2 mg/mL glucose, 80 µg/mL catalase, and 0.67 mg/mL glucose oxydase), and 1.5% BSA (Figure 1A). MT assembly was visualized using an objective-based TIRF microscope (Nikon TE2000-E) and Quantem 512SC camera (Photometrics). Microscope stage was kept warm (32°C) using a warm stage controller (LINKAM MC60). Excitation was achieved using 491 nm and 561 nm lasers (Optical Insigths). Time-lapse microscopy (one frame every 2 s) was performed for 30 min using Metamorph software (Universal Imaging).

### MT Nucleation from Axonemes

Sea urchin (*Strongylocentrotus purpuratus*) axonemes spotted on a coverslip were first mixed for 5 min with 10 µM tubulin at 35°C to initiate MT nucleation and elongation, and then perfused with various concentrations of MAP65-1. MT elongation was recorded on TIRFm as before.

### Analysis of the MT Bundle Dynamics with Kymographs

**Figure 2:**
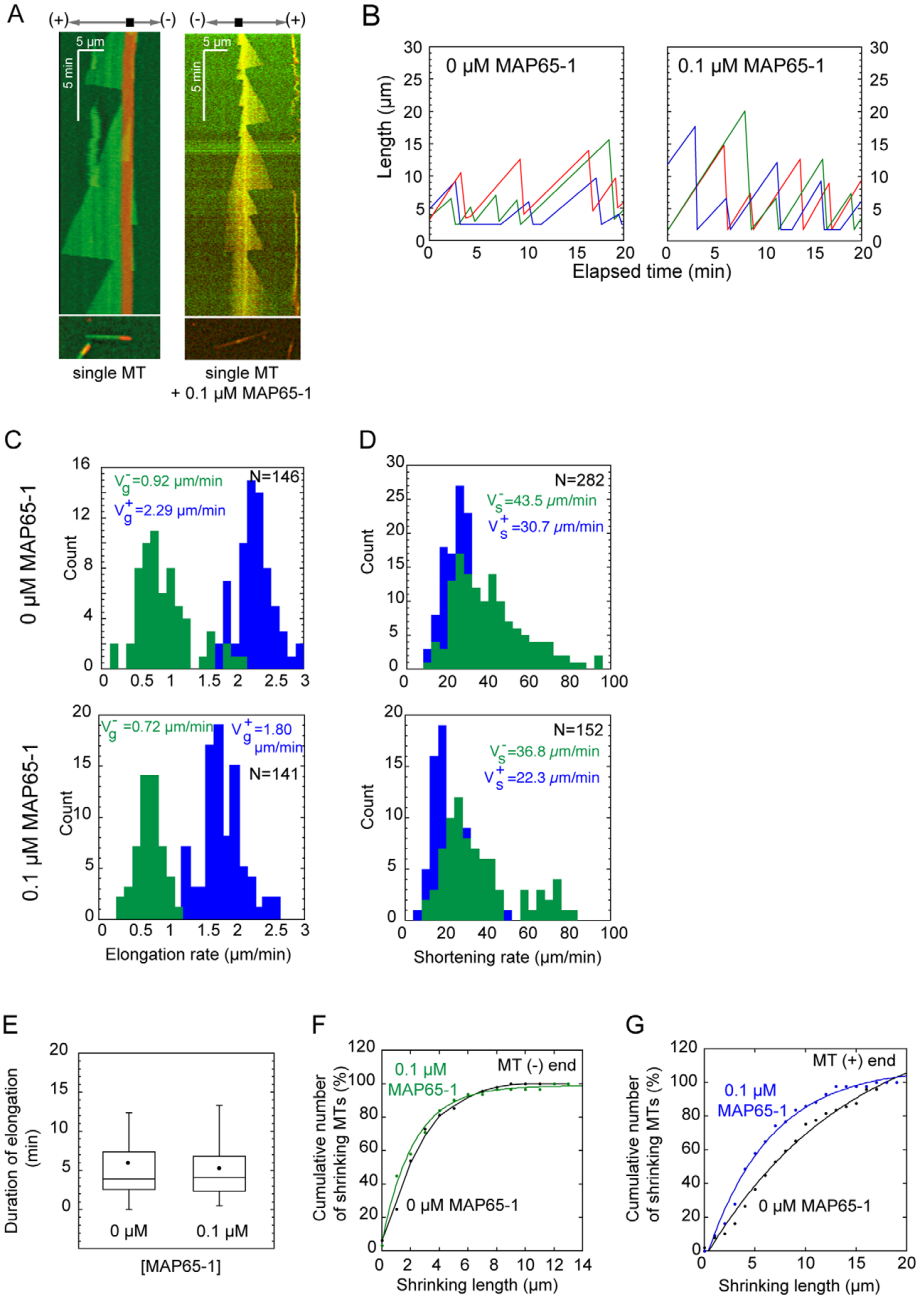
Effect of MAP65-1 on the dynamics of individual MTs. (**A**)**.** Kymographs of single MTs that elongate in the absence of MAP65-1 (left kymograph), or in the presence of 0.1 µM of GFP-MAP65-1 (right kymograph). In the first kymograph, a single Alexa-488 MT (green) elongates from an Alexa-568 MT seed (red). In the right kymograph, Alexa-568 MT (red) elongates from an Alexa-568 MT seed (red) in the presence of GFP-MAP65-1 (green). The yellow color reveals the binding of GFP-MAP65-1 on the MT. Schemes on top of the kymographs show the orientation of MT ends. Bottom images show the MTs used to draw the kymographs. (**B**)**.** Length history plots of 3 single MTs over time in the absence and in the presence of 0.1 µM MAP65-1. (**C–D**)**.** Distribution of elongation rates (C) and shortening rates (D) of single MTs in the absence (top row) or in the presence of 0.1 µM MAP65-1 (bottom row). Data for MT (+) and (−) ends are shown in blue and green respectively. Average rates and population size are indicated. (**E**)**.** Duration of elongation phases of single MTs after a rescue event in the absence and in the presence of 0.1 µM MAP65-1. Black dots indicate the mean duration of elongation phases. (**F–G**)**.** Depolymerization length of MTs in the absence and in the presence of 0.1 µM MAP65-1, for MT minus end (F) and MT plus end (G).

Time-lapse movies were treated with filters (equalize light, low pass and flatten background of Metamorph software) to improve signal/noise ratio. Kymographs of the MTs were generated using Metamorph and analyzed with ImageJ (Rasband, W.S., ImageJ, U. S. National Institutes of Health, Bethesda, Maryland, USA, http://rsb.info.nih.gov/ij/, 1997-2008). In the present study, we considered as bundle all arrays that contain at least 2 distinguishable MTs, as judged by the presence of several MT traces on the kymographs. Kymographs where several MT traces could not be unambiguously distinguished have not been considered as bundles. Elongation/shortening rates correspond to the slope of elongating/shrinking MTs inside the bundles respectively. (+) and (−) MT ends were easily identified on kymographs of single MTs and of bundles containing a low number of MTs (2–3). (+/−) MT ends correspond to fast/slow growing ends (high/low slopes on the kymographs). In complex kymographs that correspond to bundles containing numerous MTs, the traces of individual MTs cannot be follow over long period. For such bundles, rates of MTs were calculated without assigning a specific polarity to the MTs. We considered that the bimodal distribution of rates correspond to (+) and (−) ends, in respect to the bimodal distribution of single MTs (Figure 2C–D, Figure S1A–B; Figure S2B–C; Figure S3C–D).

Measurements of the dynamics of MT bundles (duration of elongation, shrinking and elongation length) were extracted from the life history plots of the corresponding kymographs (see examples in Figure 1E and S2D). Traces correspond to the longest MT of the bundle, thus illustrating the dynamic of the whole bundle. Duration of elongation corresponds to the time between rescue and catastrophe events. Length of elongation corresponds to the distance on which MTs/bundles grow between rescue and catastrophe events. Length of depolymerization corresponds to the distance on which MTs/bundles shrink between catastrophe and rescue events. Catastrophe frequency corresponds to the number of catastrophe events divided by the time MTs/bundles elongate and pause. Rescue frequency corresponds to the number of rescue events divided by the time MTs/bundles depolymerize. The length of a bundle corresponds to the length of the longest MT of the bundle. The statistical significance was determined using Student’s *t* test and non-parametric Kruskal-Wallis test.

### Modeling of MT Bundle Growth in the Presence of MAP65-1 and MAP65-4

We first simulated the kinetics of unbundled MTs, including polymerization/depolymerization rates and rescue/catastrophe frequencies to account for the dynamics of MT alone. Then we considered that MAP65 establish bonds between pairs of parallel and/or anti-parallel MTs, thereby modifying the MT bundle growth. Different models for the interactions between either MAP65-1 or MAP65-4 and MT orientation within bundles (parallel or anti-parallel) were studied to account for the average bundle growth observed in TIRF microscopy. See the supplementary material section, Figures S6 and S7 and Tables S1, S2 and S3 for a detailed description of the model.

## Results

### Dynamics of MTs in MAP65-1/4 Induced Bundles

The main question we asked in this study is whether dynamics of MTs are modified by lateral MAP65 links between MTs as it occurs in bundles. To answer this question, we analyzed the dynamics of MAP65-1 induced bundles. We used an *in vitro* system to generate bundles of growing MTs (up to 7 MTs) (Figure 1A). First, short stable MTs were bundled by MAP65 in order to generate short stable bundles that were further attached to a coverslip via biotin/neutravidin interactions. In a second step, these short bundles served as seeds to elongate MTs in the presence of tubulin (22 µM) and various MAP65 concentrations. MT bundles formation was imaged in real time using a two-color Total Internal Reflection Fluorescence microscope (TIRFm). This *in vitro* reconstitution of MT bundles allows the study of their dynamics as units with their own characteristics. Note that in the present study, most of the bundles we observed contained more than 2 MTs. This may be an important difference with previous studies where only small bundles (2–3 MTs) were examined (see [19], [22] for example).

In the assays, knowing that apparent Kd of MAP65-1 for MTs is 0.57 µM ([26]; 1.03 µM as determined in [27]), we used MAP65-1 concentrations ranging from 0.1 µM to 1.5 µM. In the presence of MAP65-1, we observed that MT bundle length increased linearly with time and generated a dense array of MT bundles (Figure 1B, Movies S1 and S2). MAP65-1 accumulates on growing bundled MTs concomitantly with the elongation of MT bundles (Figure 1C). To identify what changes are responsible for this increased assembly, we generated kymographs of bundles (length/time plots; Figure 1D). In agreement with previous observations [18], kymographs revealed a mixed MT polarity in the bundles, confirming that MAP65-1 binds anti-parallel MTs. Length history plots of MT bundles (Figure 1E) show that MT assembly increases with MAP65-1 concentrations. Analysis of these plots revealed that the elongating bundles had two main characteristics. First, the elongation duration of bundled MTs, measured as the time interval between a rescue and a catastrophe event, increased as a function of MAP65-1 concentration (Figure 1F). This MT elongation, is 2.7 min on average in the absence of MAP65-1, but jumps to an average of 6.2 min in presence of 1 µM MAP65-1. Second, MAP65-1 bundling reduced the amplitude of MT depolymerization (Figure 1G). Indeed, in the absence of MAP65-1, half of the MTs depolymerized for at least 5 µm, whereas in the presence of 1.0 µM MAP65-1 half of the bundled MTs depolymerized over 2 µm. This is easily seen in the length history plots of MTs were single MTs depolymerized up to the seed (Figure 1E, left graph), whereas shrinking bundled MTs experienced a rescue before they reach the seed (Figure 1E, three right graphs). In consequence to the limited depolymerization of MTs, the growing front of bundles was composed of MT (+) ends that elongate in close vicinity to each other, in a process we further called coordinated growth. Both, the increase of the elongation duration of bundled MTs and the reduction of the distance over which they depolymerize correlate with the age of the MTs. Indeed MT elongation increases over the whole period (Figure S1C) and the mean depolymerization length decreases with time in the presence of MAP65-1 (Figure S1D). To understand the processes that underline this collective behavior of bundles, we used the kymographs to measure the parameters of the dynamics of individual MTs inside bundles. MT elongation and shortening rates were calculated from the slopes of MT traces (see the right kymograph in Figure 1D for example). We observed that growth and shortening rates of MTs cross-linked by MAP65-1 were similar to those determined for single MTs (Figure S1A and S1B). This suggests that the increased growth of bundled MTs is due to changes in their catastrophe and/or rescue frequencies in comparison with single MTs. We observed that the apparent rescue frequency of individual bundled MTs increases with MAP65-1 concentrations (Figure 1H). We did not experimentally detect a significant change in their apparent catastrophe frequency (Figure 1I). Note that these data have two limitations. First slowly growing MTs such as MT (−) ends were embedded in the center of bundles and were hardly detectable in kymographs (Figure 1D). Therefore it is probable that MT (−) ends are underrepresented in our analysis. Second, bundles in which individual MTs can unambiguously be traced over a long period are seldom, because of the complexity of bundles. This contributes to the large errors bars in Figures 1H and 1I. From all the experimental data we obtained, we conclude that MAP65-1 bundles are characterized by long elongation periods and short depolymerization phases. This collective behavior is correlated the increase of the apparent rescue frequencies of individual MTs inside bundles. This new dynamical regime of the whole bundle amplified with time, such that depolymerization length becomes shorter and shorter as the bundle ages.

We asked whether the new dynamical regime we observed also applies to bundles with a low number of MTs. Since our experimental set-up disfavor the formation of small bundles, we could not collect enough data for reliable statistical analysis. However our measurements indicate no significant correlation between the number of MTs and the bundle growth rate.

The sustained growth of MT bundles was not specific to MAP65-1 induced bundles, as we observed that MAP65-4 bundled MTs have similar dynamics as those bundled by MAP65-1 (Figure S2; movie S3; [17]). Mainly MT elongation/shortening rates are not affected (Figure S2 A-C) and elongation/shortening amplitudes increased/decreased respectively (Figure S2 D-H).

### Dynamics of Single MTs in the Presence of MAP65-1

We then asked if the persistent growth of bundled MTs was either due to (i) the binding of MAP65 on the MT, or due to (ii) the MAP65 links between adjacent MTs. To answer this question, we compared the dynamics of single MTs in the absence of MAP65-1 (naked MTs) with the one of single MTs saturated with MAP65-1. Indeed we assumed that single MTs saturated with MAP65-1 are very similar to bundled MTs whose surface is covered with MAP65-1 that cross-link them. In our experimental set-up, maximal coverage of single MTs assembled from 22 µM tubulin was obtained with 0.1 µM MAP65-1. Indeed, when single MTs were polymerized with higher MAP65-1 concentrations, elongation of MTs was impaired and protein aggregation occurred. Therefore it is probable that 0.1 µM did not bind individual MTs with a 1∶1 stoichiometry, as it is observed in bundles [18], [19]. At the concentration of 0.1 µM MAP65-1, we observed that MAP65-1 binds along MTs (Figure 2A), and that length history plots of single MAP65-1 saturated MTs were similar to those of naked MTs (Figure 2B). Analysis of the dynamics of single naked MTs and single MAP65-1 saturated MTs reveals no difference (i) in MT shortening rates (Figure 2D), (ii) in the duration of MT elongation (Figure 2E), and (iii) in the MT depolymerization amplitude (Figure 2F and 2G). MT elongation rates were slightly reduced in the presence of 0.1 µM MAP65-1 (Figure 2C), also this was not observed in the presence of 50 nM MAP65-1 (Figure S3). In correlation with these results, there was no statistical difference between the frequencies of rescue and catastrophe events of naked MTs and MTs assembled in the presence of 0.1 µM MAP65-1 (Table 1). Thus we conclude that MAP65-1 does not affect significantly the dynamics of unbundled MTs.

**Table 1:** Frequencies of catastrophe and rescue events of single MTs in the presence of MAP65-1.

	0 µM MAP65-1	0.1 µM MAP65-1
**f_cat_ (+) end**	0.131±0.074 N = 30; t = 578 min	0.146±0.080 N = 14; t = 415 min
**f_cat_ (−) end**	0.168±0.108 N = 20; t = 378 min	0.114±0.047 N = 10; t = 314 min
**f_res_ (+) end**	2.100±1.020 N = 7; t = 578 min	2.007±0.832 N = 6; t = 415 min
**f_res_ (−) end**	9.783±3.252 N = 14; t = 378 min	7.039±3.223 N = 8; t = 314 min

Frequencies of catastrophe (f_cat_) and rescue (f_res_) of single MTs in the absence (left column) and in the presence of 0.1 µM MAP65-1 (right column) are given for MT (+) and (−) ends (mean frequency ± S.E.). N = number of values; t = time of analysis.

### Modeling MT Bundle Dynamics

Our *in vitro* experimental setup gave only a macroscopic view of the growth of MT bundles. To fully understand the mechanisms that drive the sustained growth of bundled MTs, we should follow individual MTs dynamics inside bundles. In this attempt we set up a microscopic kinetic model in which we could quantify the dynamic parameters of bundled MTs, count the number of MTs in the bundles and correlate their dynamics with MAP65 properties.

**Figure 3:**
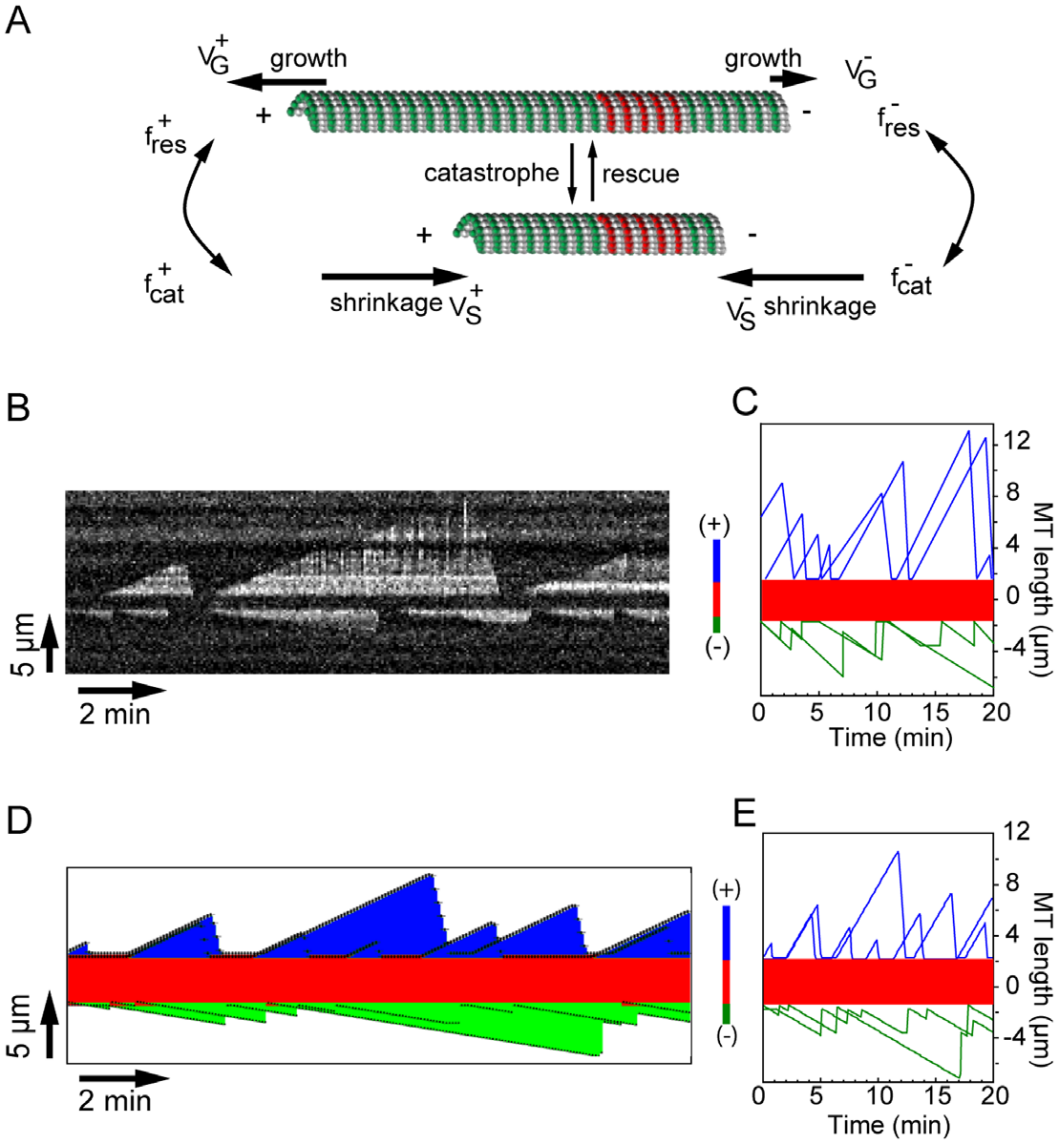
Modelisation of the dynamics of individual MTs. (**A**)**.** Scheme of a dynamic MT (green) elongating from a stable MT seed (red). The different parameters of MT dynamics are (i) growth rates at (+/−) ends (V_G_^+^/V_G_^−^), (ii) shrinkage rates (V_s_^+^/V_s_^−^), (iii) catastrophe/rescue frequencies at MT (+) ends (f_res_^+^/f_cat_^+^) and (−) ends (f_cat_^−/^f_res_^−^). (**B**)**.** Experimental kymograph of an individual MT fluctuating away from the seed (dark band at the kymograph center). Scheme on right of the kymograph shows the orientation of MT ends. (**C**)**.** History plots of two individual MTs observed by TIRFm. Blue and green traces correspond respectively to MT (+) and (−) ends. (**D**)**.** Kymograph of two simulated independent MTs elongating from a seed (red). MT (+) ends are in blue; MT (−) ends are in green. (**E**)**.** Corresponding fluctuation plots over time (same code color as in C).

**Figure 4:**
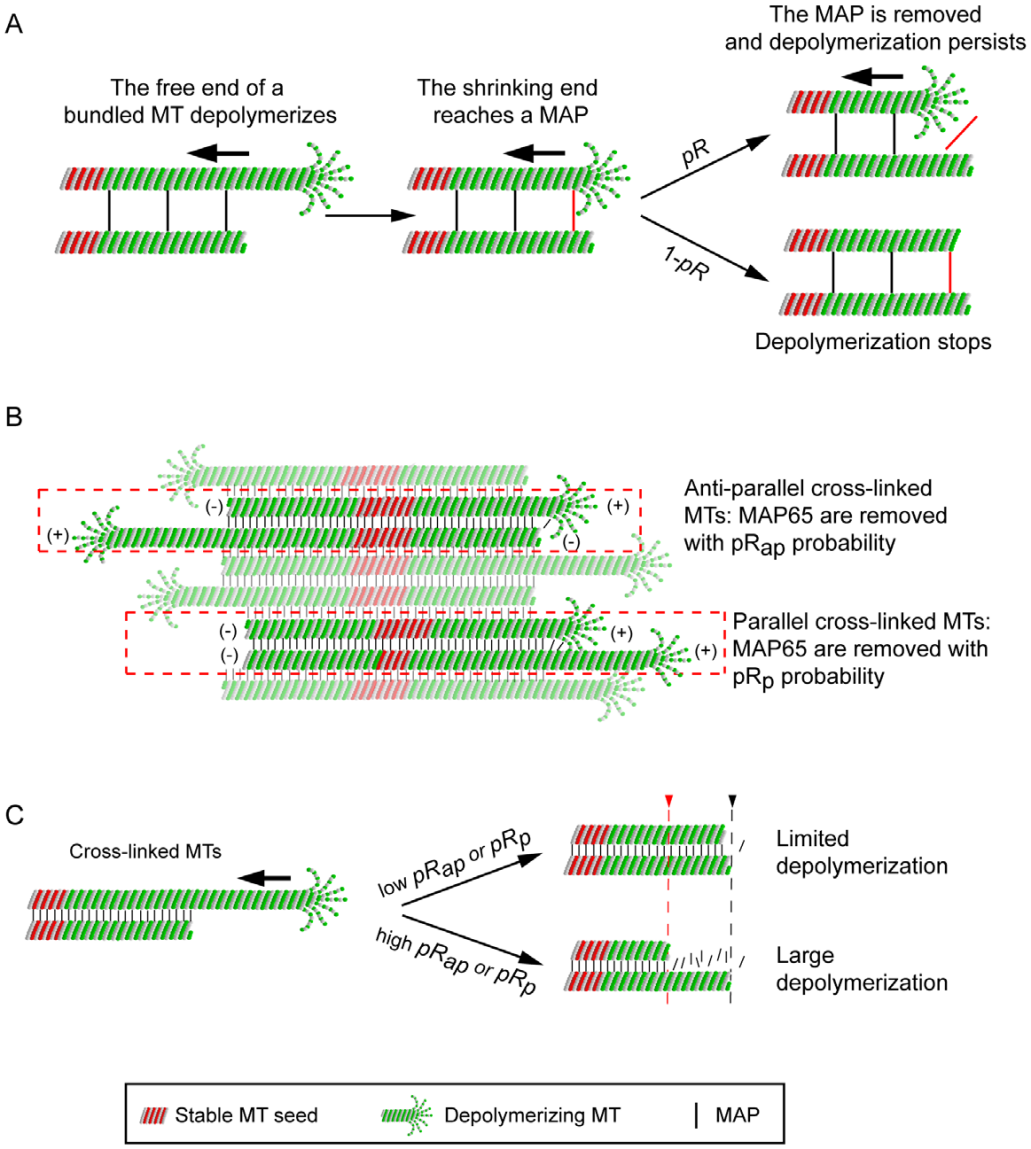
A microscopic model for the growth of MTs in bundles. (**A**)**.** Rationale used to model the effect of a MAP65 bond on MT depolymerization. Stable MT seeds are in red, dynamic MTs are in green. For sake of simplicity, only one pair of MTs cross-linked by 3 MAP65 (bars) is shown. The upper MT end undergoes a catastrophe event (left panel) and MT depolymerizes until it reaches a MAP65 bond (shown in red) at the end of the cross-linked MT (middle panel). At this stage, the MAP65 either detaches from MT with probability *pR* (right, top panel), or stays bound to the MTs with a probability (1-*pR*) (right, bottom panel). In the first case, the MAP65 bond is removed and the MT continues to shrink. In the second case, the MAP65 bond resists MT depolymerisation, which stops. (**B**)**.** During depolymerization, MAP65 (black bars) detaches from anti-parallel MTs with a probability *pR_ap_*, or detach from parallel bundled MTs with a probability *pR_p_*. The color code is the same as in (A). (**C**)**.** Effect of the value of *pR_ap_* and *pR_p_* on the amplitude of MT depolymerization. The upper MT end undergoes a catastrophe event and MT depolymerizes (left panel) before it reaches the end of a cross-linked MT (right panel). At low *pR_ap_* or *pR_p_* (top panel), most of the MAP65 molecules stay bound to the MTs. Therefore MT depolymerization is stopped at the vicinity of the end of the adjacent MT (black broken line). If *pR_ap_* or *pR_p_* is high (labile MAP bonds; bottom panel), MT depolymerization may persist behind the end of a cross-linked MT, thus generating short bundles (red broken line).

**Figure 5:**
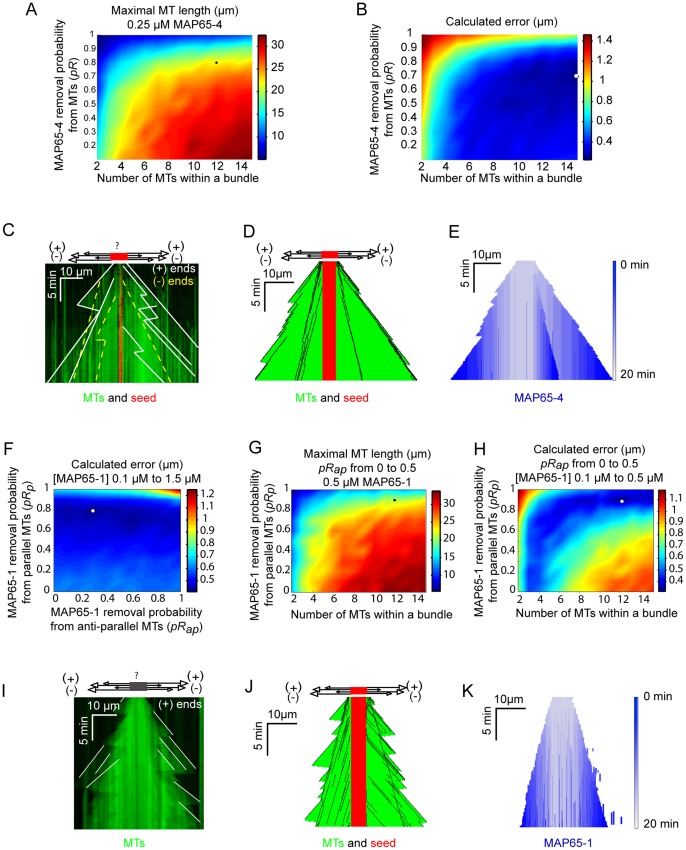
Growth of MTs bundles is dependent on the lifetime of MAP65 links. Results of MT growth simulation in the presence of MAP65-4 (A-E) and MAP65-1 (F-K). (**A**)**.** Diagram showing the predicted maximal length of MT bundles that elongate for 20 min in the presence of 0.25 µM MAP65-4, as a function of the number of MTs in the bundle (horizontal axis) and removal probability *pR* (vertical axis). Predicted bundle length (µm) is coded by the color bar on the right. Black dot indicates the conditions at which predicted and experimental bundle have the closest lengths (explained in Figure S6). (**B**)**.** Diagram of the error between calculated and measured maximal bundle length for different combinations of the number of MTs in the bundle (horizontal axis), removal probability *pR* (vertical axis), and for all available MAP65-4 concentrations. The error (µm) as coded by the color bar on the right, presents a marked minimum for a number of MTs above 10 MTs/bundle and a removal probability *pR* about 0.7. (**C**)**.** Experimental kymograph of MTs (green) bundled by 0.25 µM MAP65-4. White plain lines indicate MT (+) ends; yellow dotted lines indicate MT (−) ends. (**D–E**)**.** Kymographs of simulated bundles showing the dynamics of MTs (D) and the MAP65-4 binding (E) in the same conditions as in (D). The blue color represents the MAP65-4 binding wave as the MT elongation progresses. (**F**)**.** Diagram of the error between calculated and measured maximal length of bundles, for different combinations of *pR_ap_* and *pR_p_*. Simulations were made with MAP65-1 concentrations ranging from 0.1 µM to 1.5 µM, and the number of MTs was kept fixed at 10. (**G**)**.** Predicted maximal bundle length for different combinations of the MT number in the bundle and removal probabilities of MAP65-1 connecting parallel MTs (*pR_p_*). The diagram was obtained using a MAP65-1 concentration of 0.5 µM and removal probabilities *pR_ap_* in the range [0, 0.5]. (**H**)**.** Diagram of the error between calculated and measured maximal bundle length for different combinations of the number of MTs in the bundle (horizontal axis) and removal probability from parallel MTs *pR_p_* (vertical axis). We used MAP65-1 concentrations in the range from 0.1 µM to 0.5 µM and the removal probabilities of MAP65-1 connecting anti-parallel MTs *pR_ap_* were taken in the interval [0, 0.5]. The error as coded by the color bar on the right, presents a marked minimum for a number of MTs above 10 MTs/bundle and a removal probability *pR_p_* around 0.9. (**I**)**.** Experimental kymograph of MTs (green) bundled by 1 µM MAP65-1. White plain lines indicate MT (+) ends. Note that (−) ends are buried in the kymograph. (**J–K**)**.** Kymographs of simulated bundles showing the dynamic of MTs (F) and the MAP65-1 binding (G) in the same conditions as in (E). The blue color represents the MAP65-1 binding wave as the MT elongation progresses.

In a first step, we simulated the dynamics of MTs that elongate from individual stable seeds using the dynamic parameters extracted from kymographs corresponding to individual MTs observed *in vitro* (Table 2; Figure 3A–C; Figure S4; movie S1). Results of the simulations are illustrated by kymographs of single MTs, as shown in Figure 3D–E and movie S4. Next, we simulated the assembly of MTs bundled by both MAP65-1 and MAP65-4. To account for the reduction of depolymerization length of bundled MTs observed *in vitro* (Figure 1G and Figure S2F; [17]), we hypothesized that the MAP65 bonds between MTs in a bundle act as a stop during MT shrinkage. When a MAP65-crosslinked MT undergoes depolymerisation, it shortens until the shrinking end reaches a MAP65. At this stage, either the MAP65 bond is removed, and MT depolymerization continues, or the MAP65 bond resists and MT depolymerization stops. The resistance of MAP65 bonds to depolymerization relies on multiple parameters such as the affinity of MAP65 for MT, the affinity between MAP65 dimers, the structural conformation of MAP65, or the density of MAP65 molecules on MTs. However, this study and available data from literature are not sufficient to test all these parameters independently. Thus we considered a lumped parameter to model the resistance of a MAP65 to MT depolymerization (Figure 4A). In the model, a MAP65 bond has a probability *pR* to be removed by a depolymerizing MT, and a probability (1-*pR*) to resist MT depolymerization and stop it. In addition, we consider that MAP65 connecting anti-parallel or parallel MTs inside a bundle are removed with probabilities *pR_ap_* and *pR_p_*, respectively (Figure 4B). Thus in our model the amplitude of MT depolymerisation depends on the MAP65 sensitivity to MT depolymerization (Figure 4C). On one hand, low *pR_ap_* (or *pR_p_*) *(*close to 0) limits the depolymerisation to short distances. On the other hand, with high *pR_ap_* (or *pR_p_*) *(*close to unity), MT-MT MAP65 bonds are easily removable and depolymerization extension is almost independent of the presence of MAP65. We simulated the MT dynamics in MT bundles for different values of *pR_ap_* and *pR_p_*, and for different numbers of MTs in the bundles. The accuracy of the model is evaluated by the difference between the expected maximal MT bundle length given by the model and the MT bundle length observed *in vitro* over a period of 20 minutes (Figure 5B, F and H). The distance between experiment and model is represented as a color map, and is explained in Figure S6. We estimated *pR_ap_* (or *pR_p_*) and the number of MTs in bundles as the parameter combination that minimizes this error for all tested MAP65 concentrations.

**Table 2:** Experimental values used to simulate the dynamics of individual MTs.

MT	Growth rate*(µm/min)*	Shrinkage rate*(µm/min)*	Catastrophe rate*(events/min)*	Rescue frequency*(events/min)*
Plus end *(1451 min)*	1.56±0.012 (N = 245)	14.25±0.276 (N = 145)	0.236	0.091
Plus end *(1599 min)*	0.56±0.006 (N = 271)	40.49±1.071 (N = 153)	0.197	0.172
**MT**	**Pause frequency ** ***(events/min)***	**Growth time ** ***(min)***	**Shrinkage time ** ***(min)***	**Pause time ** ***(min)***
Plus end *(1451 min)*	–	3.56±0.118 (N = 359)	0.56±0.006 (N = 271)	–
Plus end *(1599 min)*	0.012 (N = 21)	4.75±0.209 (N = 273)	0.26±0.048 (N = 259)	1.84±0.309 (N = 19)

Kymographs and length history plots of simulated MTs are shown in Figure 3D and 3E.

### MT Polarity Control Bundle Growth

We first consider a simple situation in which the polarity of bundled MTs is not a discriminant parameter to control bundle growth. This should be the case for bundles that elongate in the presence of MAP65-4, as MAP65-4 randomly cross-links parallel and anti-parallel MTs [17]. In this situation, we impose that *pR_ap_* and *pR_p_* have the same value, *pR*. Our model was sufficient to generate MT bundle whose lengths were comparable to those observed experimentally for different MAP65-4 concentrations (Table 3). We determined that the combination of at least 6 MTs with a *pR* of 0.7 gave the best match between the model and the experiments (Figure 5B). If *pR* <0.7, then the predicted MT length is larger than those measured *in vitro* (Figure 5A). Using these parameters (*pR* = 0.7; 10 MTs), predicted kymographs of MT bundles are very similar to their experimental counterparts (Figure 5C–E; movie S5). Note that kymograph similarity is measured by the difference between measured and simulated bundle length (Figure S6). They clearly show that bundles have a sustained growth, and that depolymerization phases are short. Kymograph of bound MAP65-4, given in Figure 5E, illustrates the residence time of MAP65-4 on bundled MTs. This kymograph shows that MAP65-4 accumulates in the bundles as MTs elongate, although MAP65-4 bonds are labile. Altogether these data are in agreement with recent experimental data that indicated the non-selectivity for MT polarity of MAP65-4 [17].

**Table 3:** MT length in the presence of MAP65-4.

[MAP65-4] (µM)	observed length (µm)	predicted length (µm)
0.00	7.5±4.7	7.54
0.10	10.1±4.1	10.3
0.25	18.3±7.2	22.0
0.50	26.4±14.8	32.1

Comparison of the experimentally observed MT maximal length (left column) and the MT maximal length predicted by the model (right column). Data indicate the mean value ± S.E.

We next ask how MT orientation inside bundles affects the growth of the bundles induced by MAP65-1. Since previous studies have shown that MAP65-1 strongly selects anti-parallel MTs [18], we impose that *pR_ap_* is independent from *pR_p_*. In a first step, we assumed that MAP65-1 connects anti-parallel MTs only (i.e. *pR_p_* is set to 0). Using this hypothesis, we observed that the maximal MT length in the bundle is systematically underestimated by the model (Table 4), resulting into a large error. Because MTs coupling comes in pairs, the growth of MT ends by MAP65-1 is limited by the growth rate of the slowest growing MT end of the pair, i.e. the minus end in anti-parallel MT pairs. Thus we relaxed the assumption that MAP65-1 forms anti-parallel MT pairs only, and we introduced the possibility that MAP65-1 makes bonds between parallel MTs (i.e. *pR_p_* ≠ 0, Figure 4A). To evaluate the respective effects of MAP65-1 links between parallel MTs versus anti-parallel MTs, we simulated the growth of bundles of 10 MTs for different combinations of *pR_ap_* and *pR_p_*. As before, we compared the maximal length of simulated bundles to the length of experimental MT bundles. Computed errors for all MAP65-1 concentrations revealed that the best fit was obtained for high *pR_p_* (∼0.8) and low *pR_ap_ (*∼0.3) (Figure 5F). This result indicates that MAP65-1 bonds between parallel MTs are easily removed during MT depolymerization. It also suggests that although MAP65-1 bonds between parallel MTs are rather loose, they have a major effect on the length of the MT bundle. This makes sense as bonds between parallel MTs allow (+/+) MT ends connections that can readily elongate. If these bonds were absent or were too labile (*pR_p_* close to 1), MT bundles would be shorter than those observed *in vitro* (Table 4; Figure 5F). If they were too stable (low *pR_p_*), then MT bundles would be too long. In a second step, we varied the number of MTs and *pR_p_,* and we calculated the length of MT bundles for a fixed *pR_ap_* value chosen in the interval [0, 0.5]. Simulations were carried out for different MAP65-1 concentrations (0.1 µM; 0.15 µM; 0.5 µM) (Figure 5G; see also Figure S5A for a systematic evaluation of the bundle dynamics for different *pR_ap_*). Best parameter fits were obtained for simulated bundles with more than 6 MTs and high *pR_p_* (around 0.9) (Figure 5H), whatever the actual value of *pR_ap_* or the concentration of MAP65-1. This confirms that MAP65-1 bonds between parallel MTs are labile (high *pR_p_*); conversely, the error rapidly increases for low *pR_p_* (Figure 5H) because the simulated MTs are too long compared to the experimental ones (Figure 5G). These data corroborate the hypothesis that bonds between parallel MTs have a strong effect on MT length. In bundles having more than 6 MTs, this property is independent of the sensitivity of MAP65-1 links between anti-parallel MTs (*pR_p_*; Figure S5B), and of MAP65-1 concentration (Figure S5C). The predicted kymograph of MAP65-1 cross-linked MTs (Figure 5J, Movie S6) is similar to the experimental one (Figure 5I; Movie S2). This kymograph shows that leading MT (+) ends control the size of the bundle. Although MAP65-1 bonds can be easily removed, as indicated by the blue isolated segments (Figure 5K; movie S6), their effect is sufficient to protect parallel MTs from large depolymerization. Depolymerization of MT (+) ends is also blocked by MT (−) ends, as rescue events occurred in the vicinity of the MT (−) ends (Figure 5J).

**Table 4:** MT length in the presence of MAP65-1.

[MAP65] (µM)	Observed length of bundled MTsof mixed polarity (µm ± SD)	Predicted length of bundled MTsof mixed polarity (µm)	Predicted length of anti-parallel bundled MTs (µm)
0.00	7.5±4.7	7.54	
0.10	15.5±8.7	18.6	9.97
0.15	16.2±6.8	18.1	
0.25	23.9±8.7	24.0	17.6
0.50	20.9±6.5	23.6	
0.75	18.2±7.8	19.1	
1.00	26.4±11.7	29.0	18.3
1.25	24.9±8.6	25.2	
1.50	27.0±4.7	28.8	

Comparison of the experimentally observed MT maximal length (left column) and the MT maximal length predicted by the model (middle column). The right most column gives the predicted MT maximal length in MAP65-1-stabilized bundles assuming connections between anti-parallel MTs only. In this case, the calculated maximal length is systematically lower than the experimental one.

To validate that MAP65-1 has the ability to bundle parallel MTs as required by the model, we nucleated MTs from axonemes, thus creating an array of close parallel MTs. In the absence of MAP65-1, nucleated MTs radially organize from the axonemes, whereas in the presence of MAP65-1, they form a thick bundle (Figure S5D). Thus MAP65-1 also binds parallel MTs *in vitro*. Furthermore, we performed FRAP experiments on MAP65-1 mediated bundles, containing either parallel and anti-parallel MTs, or parallel MTs only (axoneme-nucleated MTs bundled with MAP65-1). Mean half-time recovery of MAP65-1 on parallel MTs was 25.4 sec (from 3.8 sec to 61 sec; N = 18), whereas it varies from 3.5 sec to 231 sec (N = 29) on bundles of mixed MT polarity. The range of half-time recovery, especially for bundles of mixed MT polarity, may result from the variability in the number of MTs present in each bundle, as this parameter influences the diffusion of MAP65-1 molecules between MTs. Nevertheless this observation suggest that MAP65-1 cross-links between parallel MTs are labile. These observations are in agreement with a recent study showing that the dwell-time of MAP65-1 on parallel MTs is larger than its dwell-time on anti-parallel MTs [27]. Altogether these data demonstrate that in bundles, MAP65-1 binds to both parallel and anti-parallel MTs. But the MAP65-1 links between parallel MTs are more sensitive to MT depolymerization than those between anti-parallel MTs. The stability of MAP65-1 bonds between parallel and anti-parallel MTs control the length of MT bundles.

### MT Dynamics Inside Bundles

**Figure 6:**
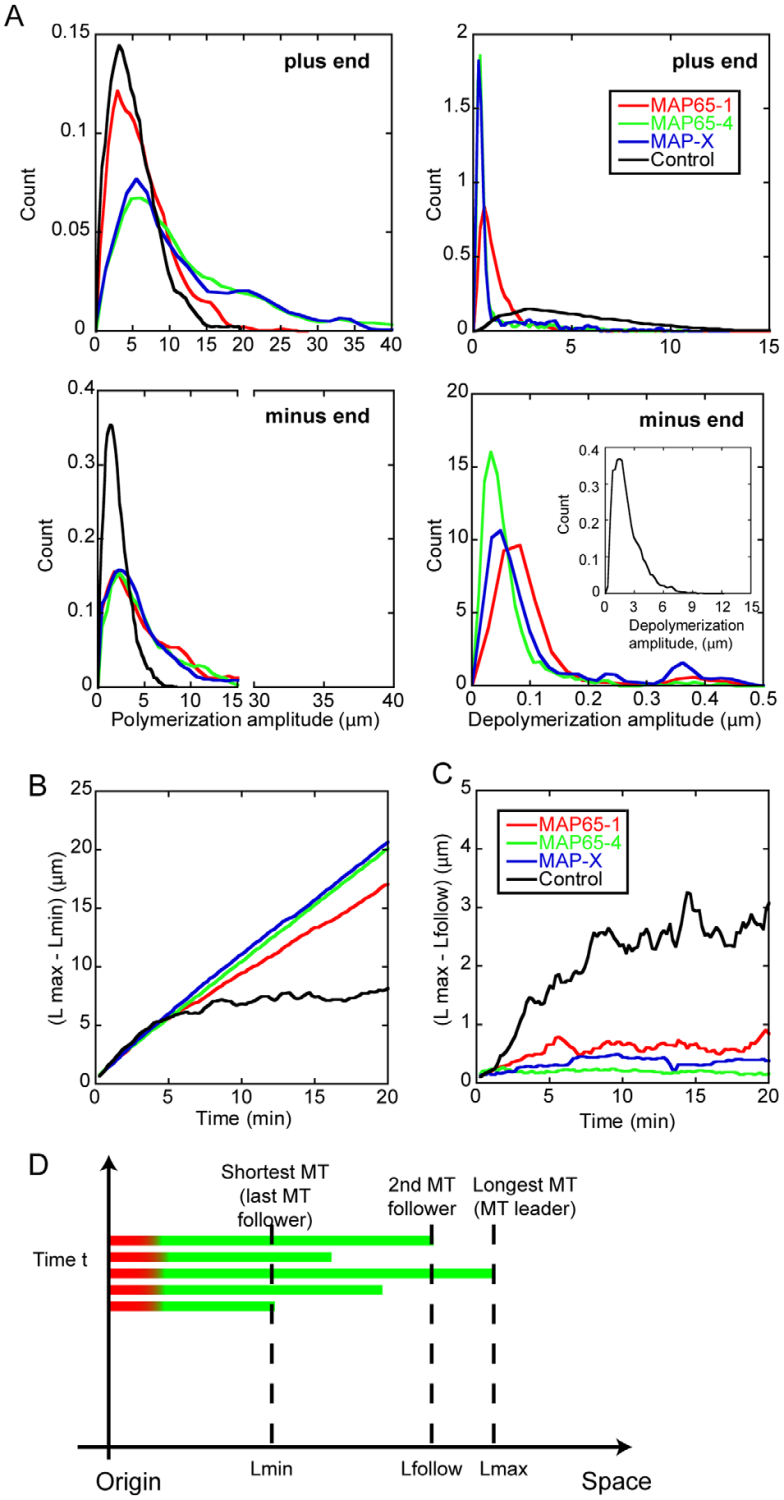
Model predictions for MT dynamics in bundles in the presence of MAP65. Simulations were performed with MAP65 binding rates of k_on_ = 10 µM.s^−1^, k_off_ = 0 s^−1^, and MAP65 concentration of 0.1 µM; the number of MTs in the bundle is 10. The MAP65 removal probabilities are, for MAP65-1, *pR_ap_* = 0.1 and *pR_p_* = 0.8, for MAP65-4, *pR_ap_* = *pR_p_* = 0.7, and for MAP-X *pR_ap_* = 0.8 and *pR_p_* = 0.1. In marked contrast with MAP65-1, the hypothetical MAP-X favors pairs of parallel MT (low *pR_p_* and high *pR_ap_*). In control conditions, we have *pR_ap_* = *pR_p_* = 1. Each distribution curve corresponds to 20 independent model runs. (**A**)**.** The distribution of polymerization length (left column) or depolymerization length (right column) is shown for all (+, top row) and (-, bottom row) MT ends in the bundle. The inset shows the depolymerization of the control bundle. Note the change in the scale of the abscissa axis. (**B**)**.** Average distance between the longest (leader) MT and the shortest MT (last MT follower) (L_max_−L_min_) in the bundle. (**C**)**.** Average distance between the leader MT and the next follower MT (L_max_−L_follow_). (**D**)**.** Cartoon showing how (L_max_−L_min_) and (L_max_−L_follow_) are defined. Note that the identity of the leader and follower MTs can change in the course of time. The seed and the dynamic section of each MT are shown respectively in red and in green.

To get further insight in the dynamics of MTs in bundles, we computed the distribution of polymerization/depolymerization amplitude and the rescue/catastrophe frequencies in different conditions. We used the best-fit parameters obtained for MAP65-1 (*pR_ap_* = 0.1 and *pR_p_ = *0.8) and for MAP65-4 (*pR_ap_ = pR_p_* = 0.7). In addition, we considered a hypothetical MAP, denoted MAP-X, with parameters *pR_ap_* = 0.8 and *pR_p_ = *0.1; note that MAP-X represents the “symmetric” of MAP65-1. Finally, we also simulated control conditions for which *pR_ap_ = pR_p_* = 1, *i.e.* the MAP has no effect on MT depolymerisation. Bundle growth was simulated for a period of 20 min with 10 MTs in the bundle. We used 20 independent model runs, yielding an average of 10,000 events for each distribution curve. We found that in the presence of MAP65-1, MAP65-4 or MAP-X, the polymerization amplitude of bundled MTs was enhanced for both (+) and (−) MT ends (Figure 6A, left column). In marked contrast, the depolymerization amplitude was greatly reduced for both ends (Figure 6A, right column). These results show that the 3 MAP we tested affect the dynamics of MTs in bundles. This is in agreement with the growth of a whole bundle as observed at the microscopic level *in vitro* assay (Figure 1 and Figure S2).

To characterize the growth regime of bundles, we plotted the distance between the longest (L_max_) and the shortest MT (L_min_) in a bundle during its elongation (Figures 6B and 6D). In control conditions, this distance (L_max_−L_min_) increased slowly before reaching a plateau (∼8 µm), approximately 10 min after the initial rise (Figure 6B, black curve). The existence of a plateau indicates a steady-state regime characterized by the exact balance between depolymerisation and polymerization. This is related to the distribution of polymerization and depolymerization lengths of MT (+) ends, that both peak at about the same value (Figure 6A, black curves, top row). In contrast, the presence of MAP bonds (from MAP65-1, MAP65-4 or MAP-X) yields to a sustained linear increase of the (L_max_−L_min_) distance (Figure 6B, color curves). This indicates that polymerization and depolymerisation are no more balanced inside the bundle. This observation is coherent with the distribution of polymerization and depolymerisation lengths, since their maxima occur, respectively, at ∼5 µm (polymerization, Figure 6A, top, left, color curves) and at ∼0.08 µm (depolymerisation, Figure 6A, top, right, color curves). In consequence, the average MT polymerization is favored. To further understand the dynamics of the bundle extremity, we considered the distance between the MT leader (L_max_) and the second MT follower (L_follow_), (Figure 6C and 6D). In control conditions, this distance (L_max_−L_follow_) is very similar to (L_max_−L_min_), (compare black curves, Figure 6B and 6C) showing that all the MTs are at steady state, once the initial phase has ended. In presence of MAPs, this distance (L_max_−L_follow_) raises slowly before reaching a plateau, which is almost identical for all MAPs, and which is much smaller than the plateau observed in control conditions (about 0.8 µm vs. 3 µm for control). We conclude that, in presence of MAPs, the two closest MT ends forming the bundle tip are in a “bound” regime, characterized by a highly coordinated growth.

**Figure 7:**
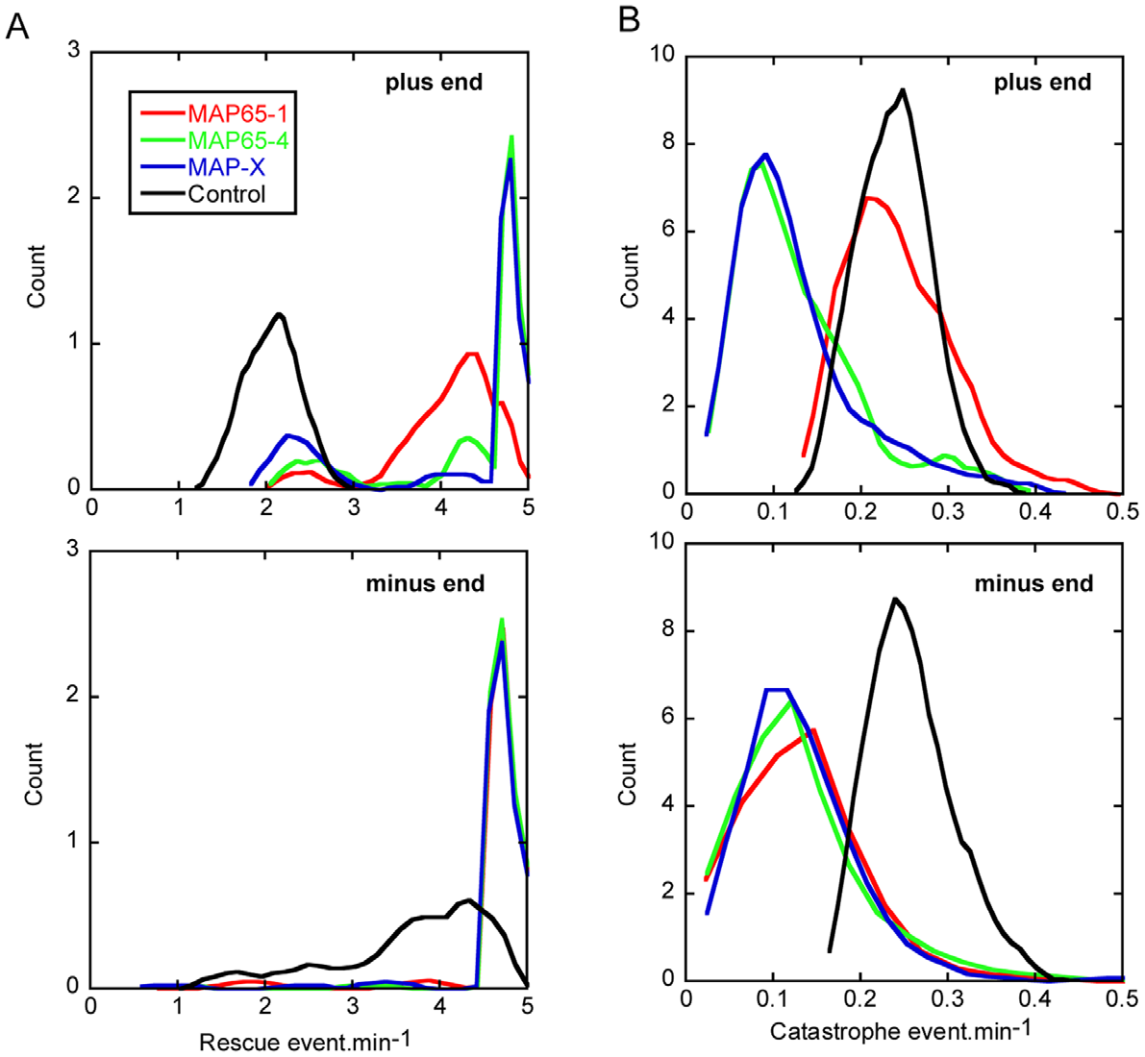
Model prediction for catastrophe and rescue frequencies in the presence of MAP65. Parameters for simulation are given in Figure 6. The distribution of rescues (A) and catastrophe (B) frequencies is shown for the plus (top row) and the minus (bottom row) MT ends in the bundle.

The reduction of the amplitude of MT depolymerization by MAP65 should induce an increase of their rescue frequencies. Indeed the model shows that in bundles, rescues are more frequent in the presence of MAP65, at both (+) and (−) ends (Figure 7A, compare black curve vs. color curves). These data are coherent with our experimental observations (Figure 1H). Interestingly, the model predicts a bimodal distribution for rescue frequencies at MT (+) ends (Figure 7A, top, color curves). The model gives a frequency of 2.5 rescue events.min^−1^ for anti-parallel bundled MTs, and a frequency comprised between 4.3-4.8 rescue events.min^−1^ for parallel bundled MTs. Moreover this bimodal distribution is observed for all MAP65, including MAP65-4, which has no selectivity for MT polarity. This implies that the differences in rescue frequencies may rely on the intrinsic dynamic properties of (+) and (−) MT ends, and not on the properties of MAP65 bonds. The model also predicts that catastrophe frequencies are decreased in the presence of MAP65 at both ends of the MTs (Figure 7B). Thus the combination of reduced catastrophe and increased rescue frequencies results into long polymerization periods and short depolymerization phases allowing MT bundles to grow steadily. Noticeably, the model predicts that MAP65-4 and MAP-X have a stronger effect than MAP65-1 (compare blue and green curves with red curves in Figures 6 and 7). Because MAP65-4 and MAP-X favor the existence of bonds between parallel MTs, these results reinforce the hypothesis that parallel bundled MTs control the growth of the bundles.

## Discussion

In this study, using combined *in vitro* and *in silico* approaches, we have shown that two plant MAP65, while bundling MTs, modify the dynamics parameters of MTs that triggers the global increase of the bundle length. Indeed, MAP65-1/4 increase the number of rescue events by reducing the distance over which cross-linked MTs depolymerize, and decrease MT catastrophe frequency so that cross-linked MTs spend more time on growing than unbundled MTs. In consequence, bundled MTs are longer than the unbundled MTs, and they display a collective behavior that is characterized by a coordinated growth of MT ends, which grow in close vicinity to each other. Conversely, MAP65 do not affect MT growth and shrinkage velocities, in agreement with observations made in living plant cells [23]. Comparable data were reported for the orthologs PRC1 and Ase1 [13], [20], [22]. The changes in the frequency of MT catastrophe and rescue events are correlated with the probability (*pR)* that a MAP65 inter-MT bond is removed during MT depolymerization. Our model predicts that even labile MAP65 bonds are sufficient to change MT dynamics. Indeed, although MAP65-4 bonds have a high probability (70%) to be removed during MT depolymerization, MAP65-4 cross-linked MTs exhibit an increase of rescue and a decrease of catastrophe events leading to sustained and coordinated growth of bundles.

Furthermore, we also observed reduced but yet detectable effects of MAP65 on MT growth at a concentration of about 0.1 µM MAP65-1 in experiments and simulations. This suggests that low concentrations of MAP65 that probably occurs in cells might be sufficient to coordinate growth of bundled MTs. In consequence, we assume that *in vivo*, MAP65 stabilizing effects may occur. It will now be important to determine the molecular events that drive the sustained growth of MTs bundled by MAP65.

Noticeably, the model predicts that MAP65 stabilizing effects are predominant in large bundle (>6 MTs). This may be an important parameter to understand the effects of MAP65 on large bundles observed in living cells. In particular, in plant cells the cortical MT arrays are composed of a heterogeneous population of MTs, from individual MTs to bundles of 2 to 8 MTs [29]–[31]. In these arrays, the effects of MAP65-1 on MT bundle growth may be rather weak on small bundles, whereas stronger in large MT bundles. The more pronounced effects of MAP65 on the dynamic of large bundles in comparison with small bundles have two important consequences. First, it might explain why the biased MT dynamics induced by MAP65 has not been observed with PRC1 and Ase1 *in vitro*. In these studies, the dynamics of small bundles was analyzed (about 2 MTs; [19], [20], [22]). Indeed, as predicted by the model, the stabilization by MAP65 is less effective in small bundles. Second, because MAP65 have no significant effect on the dynamic of individual MTs, the growth of a bundled MT must result from its interactions with several adjacent MTs mediated by MAP65 links. Upon crosslinking, MAP65 might adopt a specific configuration, as suggested for PRC1 [19] that modifies the interactions between protofilaments, or changes the on- and off-rates of tubulin at the end of MTs.

As MAP65 present differences in their selectivity for MT polarity, we evaluated the role of MT polarity in the bundle growth using our model. For MAP65-1 that preferentially links MTs of opposite polarity [18], the model predicts that MAP65-1 bundles made of anti-parallel MTs only are shorter than those observed *in vitro*, and that the presence of parallel bundled MTs was necessary to enhance their growth. The fact that MAP65-1 is able to crosslink both parallel and anti-parallel MTs is consistent with other studies showing that MAP65-1 localizes *in vivo* with parallel and anti-parallel overlap MTs [16], [23]. These data correlate with studies showing that Ase1 and PRC1 have also a strong but not exclusive preference for binding anti-parallel MTs *in vitro* [19], [20], [22]. Furthermore, our model predicts that bonds between parallel MTs control the amplitude of bundle elongation, despite the fact that they are very sensitive to MT depolymerization. In agreement with these data, a recent study showed that the dwell time of MAP65-1 is three times longer on anti-parallel MTs than on parallel MTs and on single MTs [27].

What could be the *in vivo* relevance of the different lifetime of MAP65-1 links between parallel and anti-parallel MTs? The stabilizing effects of MAP65 proteins have not yet been reported *in vivo*. In bundles containing a small numbers of MTs (2 or 3), the selectivity of MAP65-1 for anti-parallel MTs would be an efficient way to sort randomly oriented MTs into anti-parallel bundles, as already suggested for PRC1 [19], or to stabilize anti-parallel MTs in the midzone of the mitotic spindle. In bundles containing a larger number of MTs with mixed polarity such as the plant cortical MTs, parallel MT pairs could modulate the growth of the bundles. These bundles would then contain two populations of bundled MTs: anti-parallel MTs in the core of the bundle, and long leading parallel (+/+) MTs that would determine the length of the bundle. It is interesting to note that a low affinity of MAP65-1 for parallel MTs, rather than a high affinity is very effective in finely tune bundle growth.

Overall, the data obtained in this study suggest that in plant cells, MAP65 influence MT growth. Up to now, only few observations of the dynamic behavior of bundled MTs have been reported *in vivo*. Recently, Shaw and Lucas [28] observed no significant differences between the catastrophe and rescue frequencies of bundled and unbundled MTs in the *Arabidopsis* cortical arrays, although the presence of MAP65 was not correlated with MT dynamics. In the same study, they also reported a smaller relative number of rescue events for bundled MTs, leaving open the possibility than MT bundling could interfere with rescue *in vivo*. In parallel, Lucas et al. [23] studied the dynamic behavior of bundled cortical MTs that are labeled with MAP65-1. These authors observed numerous catastrophe and rescue events within bundles, but the frequencies of these events could not be measured, because of the complexity of the labeling. Finally, it has been reported that MT (+) ends spend around 75% of their time in growth, punctuated by shorter depolymerizing periods [28]. These observations are reminiscent with the behavior of MTs (+) ends we observed *in vitro*. These *in vivo* observations suggest that the effects of plant MAP65 we observed *in vitro* are rather discreet phenomena *in vivo* that would occur on specific MT subpopulations or at specific stages of the auto-organization of MT arrays. Thus, in line with our observations, we propose that the stabilizing effects of MAP65 could be dominant at the leading MT ends of some bundles, creating long bundles with MT ends in close vicinity. These long bundles (10–30 µm) are observed in plant cells where they wind around the different faces [29], [32]. Because of the closeness of longest MT ends, they may be more resistant to depolymerization when encountering an obstacle [33]. In plant cells, this might explain why MAP65-1, in combination with CLASP (+TIP protein), facilitates the passage of cortical MTs through sharp cell edges [32].

Finally, we may hypothesize that the proteins which control MT end dynamics, such as +TIPs proteins or depolymerizing kinesins, would benefit from MT end closeness, as it could increase their efficiency on adjacent MTs having coordinated growth. This synergy between different mechanisms (i.e. coordinated growth of MT ends by MAP65 and binding of +TIPs or kinesin at MT ends of either cortical MTs or in kinetochores fibers, and in interdigitated MTs during mitosis) may be an efficient way to control the length of bundled MTs.

## Supporting Information

Figure S1 **Dynamic parameters of individual MTs in the presence of MAP65-1.** **(A).** Distribution of MT elongation rates for individual MTs (top panel) or within bundles in the presence of 0.5 µM MAP65-1 (middle panel) and 1.25 µM MAP65-1 (bottom panel). Data for MT (−) and (+) ends are shown in green and blue respectively. Average rates and population size are indicated. **(B).** Distribution of MT shortening rates for individual MTs (top panel) or within bundles in the presence of 0.5 µM MAP65-1 (middle panel) and 1.25 µM MAP65-1 (bottom panel). Data for MT (−) and (+) ends are shown in green and blue respectively. Average rates and population size are indicated. Note that in bundles, the density of MTs impaired the reliable detection of (+/−) MT ends depolymerization events; both ends are shown with the same color (middle and bottom panels). In particular, (−) ends were often embedded in complex kymographs, and were underrepresented in the statistical data used in the analysis, as reported shortening rates correspond mainly to MT (+) ends. **(C).** Duration of MT elongation over time in the absence and in the presence of MAP65-1. **(D).** Amplitude of MT depolymerization length over time, in the absence or in the presence of MAP65-1.
(TIF)

Figure S2 **Dynamics of MTs bundled with MAP65-4. (A).** Kymographs of MT bundles in the presence of GFP-MAP65-4. **(B).** Distribution of MT elongation rates for individual MTs (top panel) or within bundles in the presence of 0.25 µM MAP65-4 (middle panel) and 0.5 µM MAP65-4 (bottom panel). Data for MT (−) and (+) ends are shown in green and blue respectively. Average rates and population size are indicated. **(C).** Distribution of MT shortening rates for individual MTs (top panel) or within bundles in the presence of 0.25 µM MAP65-4 (middle panel) and 0.5 µM MAP65-4 (bottom panel). Average rates and population size are indicated. Data for MT (−) and (+) ends are shown in green and blue respectively. As in Figure S1, we could not reliably distinguish (+) and (−) ends depolymerization events in dense bundles, and both ends are shown with the same color (middle and bottom panels). **(D).** Length history plot of 3 single MTs in the absence of MAP65-4 (top panel) and 3 MT bundles in the presence of MAP65-4 (middle and bottom panels). **(E).** Duration of growth phases after a rescue event as a function of MAP65-4 concentration. Dark dots indicate mean values. **(F).** Depolymerization length of MTs in the presence of MAP65-4. **(G).** Duration of MT elongation over time in the absence and in the presence of MAP65-4. **(H).** Variation of MT depolymerization length over time, in the absence or in the presence of M65-4.
(TIF)

Figure S3 **Dynamics of individual MTs in the presence of 50 nM MAP65-1. (A).** Kymographs of a single MT that elongates in the absence (left) and in the presence of 50 nM of GFP-MAP65-1 (right). Kymograph is the merge image of Alexa-568 MT (red) elongating from an alexa-568 MT seed (red) in the presence of GFP-MAP65-1 (green). Thus the yellow color reveals the binding of GFP-MAP65-1 on the MT. Schemes on top of the kymographs show the orientation of MT ends. Bottom images show the MT used to draw the kymographs. **(B).** Length history plot of 3 single MTs in the absence and in the presence of 50 nM MAP65-1. **(C–D).** Distribution of elongation rates (C) and shortening rates (D) of single MTs in the absence (left column) or in the presence of 50 nM MAP65-1 (right column). Data for MT (−) and (+) ends are shown in blue and green respectively. Average rates and population size are indicated. **(E).** Duration of elongation of single MTs after a rescue event in the absence and in the presence of 50 nM MAP65-1. Dark dots indicate mean values. **(F–G).** Depolymerization length of MT minus ends (F) and plus ends (G) in the absence and in the presence of 50 nM MAP65-1.
(TIF)

Figure S4 **Dynamic parameters of individual MTs in the absence of MAP65**. Distribution of the duration of MT elongation (**A**), the duration of MT shrinkage (**B**) and the duration of MT pause (**C**). Data for MT plus and minus ends are shown in blue and green respectively. Population size (N), mean value (m) and standard error (SE) are indicated.
(TIF)

Figure S5 **Binding of MAP65-1 to parallel MTs. (A).** Examples of calculated MT maximal length at t = 20 min, expressed as a function of *pR_p_* (removal probability for MAP65-1 connecting parallel MTs) and the number of MTs. Simulations were repeated for different values of *pR_ap_* and for a MAP65-1 concentration of 0.5 µM. **(B–C).** Optimal value for *pR_p_* as a function of the number of MTs in the bundle. We determined these values for different removal probability for MAP65-1 connecting anti-parallel MTs (*pR_ap_*) (B) or various MAP65-1 concentrations (C). Both panels show that *pR_p_* is in the range 0.9-1 and is independent of *pR_ap_* and MAP65-1 concentration. **(D).** Binding of MAP65-1 (b) to parallel MTs (a) nucleated by axonema (arrowhead). (c) is the merge image of (a) and (b). (d) Kymograph of a MT bundle as in (b). All MT elongation rates are similar (plain lines), showing that MTs are parallel. (e) MTs nucleated from an axonema in the absence of MAP65-1. MTs split apart from the axonema.
(TIF)

Figure S6 **Model simulations and comparison with experiments**. The model used in this study depends on kinetic parameters (k_on_, k_off_) of MAP65 binding, MAP65 concentrations [MAP65], number of MTs in the bundle and probability factors, *pR_ap_* and *pR_p_*, that govern the MAP65 dynamics during MT depolymerization. Of all these parameters, only (k_on_, k_off_) and the MAP65 concentrations are known. We determined the probability factors and the number of MTs that give the best fit with the experiments using the following algorithm: 1. Using a couple of parameters values (p_1_, p_2_), we simulated a series of 10 independent kymographs. 2. Then, for each simulated kymograph, we compute, Δ, the distance between the model and the experiment by summing up the difference between the predicted bundle length (L_M_(t)) and the measured length (L_E_(t)) for all available time points in both the simulated and experimental kymographs. 3. We code the value of the Δ so that the best match (Δ = 0 or minimum) is dark blue and the worst match is bright red. 4. We color the pixel at position (p_1_, p_2_) using the color corresponding to the value of Δ. 5. We repeat the procedure for all pixels in the parameter plane. The parameter combination given the best model-experimental match is directly read out from the position of the dark blue pixel(s) in the parameter plane.
(TIF)

Figure S7 **Determination of the spatial domain accessible to MAP65 connections between two microtubules. (A).** In the model, we limited our attention to MAP65 connecting MTs in the spatial domain limited by the position X(i,j,1) (left point) and X(i,j,2) (right point). The seed is indicated in red; tubulin in green. **(B).** Computation of the limit positions X(i,j,1) and X(i,j,2) for parallel (top) or anti-parallel (bottom) MTs using formulae (12–15) in the supplemental text.
(TIF)

Table S1 **List of variables and parameters used in the model for MT dynamics.**
(DOCX)

Table S2 **Matrix Transition.**
(DOCX)

Table S3 **List of parameters used in interactions between MAPs and MTs.**
(DOCX)

Movie S1 **Movies of elongating MTs in the absence of MAP65 (0.2 fps).**
(MOV)

Movie S2 **Movies of elongating MT bundles in the presence of MAP65-1 (0.5 fps).**
(MOV)

Movie S3 **Movies of elongating MT bundles in the presence of MAP65-4 (0.2 fps).**
(MOV)

Movie S4 **Simulations of MT dynamics in the absence of MAP65 (0.1 fps).** Top panel shows dynamic MTs undergoing rescues and catastrophes. MT seeds are in red, tubulin is in green; (+) and (−) indicate plus and minus MT ends respectively. Bottom. Kymographs of tubulin (left, green traces) are shown.
(MOV)

Movie S5 **Simulations of MT dynamics in the presence of 0.25 µM MAP65-4 (0.1 fps)**. Top panel illustrates MT dynamics and the control of depolymerization by MAP65-4. MT seeds are in red, tubulin is in green. MAP65-4 bonds are symbolized by blue lines connecting the MTs. (+) and (−) indicate (+) and (−) MT ends respectively. On the bottom of the Figure, kymographs of tubulin (left, green traces) and MAP65-4 (right, blue traces) are shown. The MAP65-4 binding wave is coded by the intensity of the blue color used to represents bonds (Top panel) or MAP65-4 kymographs (bottom right panel).
(MOV)

Movie S6 **Simulations of MT dynamics in the presence of 1 µM MAP65-1 (0.1 fps).** On top are shown dynamic MTs that undergo rescues and catastrophes. MT seeds are in red, tubulin is in green. MAP65-1 bonds are shown in blue. (+) and (−) indicate plus and minus MT ends respectively. On the bottom of the Figure, kymographs of tubulin (left, green traces) and MAP65-1 (right, blue traces) are shown. Same code color for the MAP65-1 binding wave as in Movie S6.
(MOV)

Methods S1 (DOCX)
