# Supplementary figures and images for: MAP65 Coordinate Microtubule Growth during Bundle Formation
Source: PLoS One. 2013 Feb 21;8(2):e56808. doi: 10.1371/journal.pone.0056808 (PMC3578873; doi:10.1371/journal.pone.0056808)

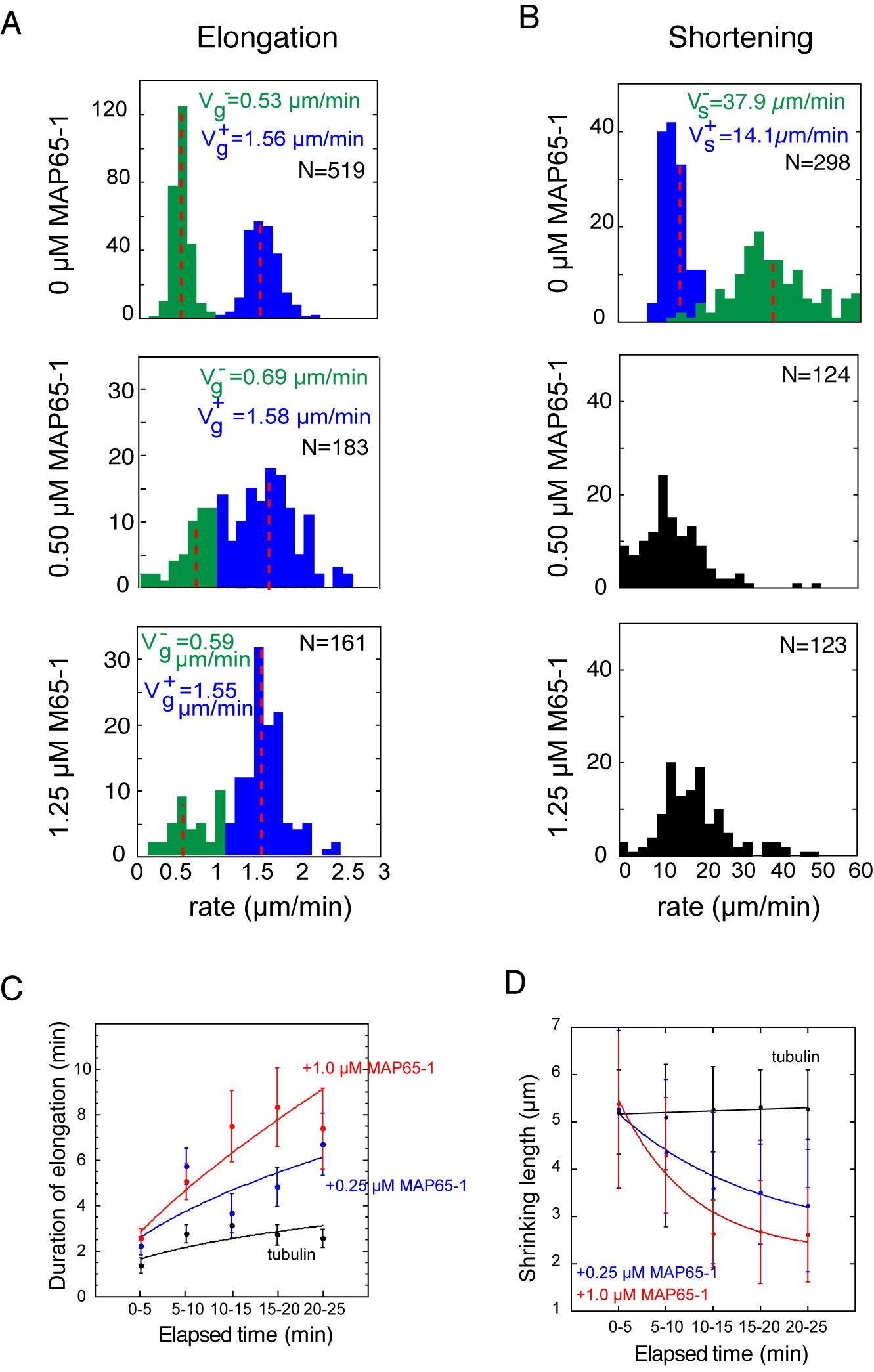

Supplement: Figure S1 — Dynamic parameters of individual MTs in the presence of MAP65-1. (A). Distribution of MT elongation rates for individual MTs (top panel) or within bundles in the presence of 0.5 µM MAP65-1 (middle panel) and 1.25 µM MAP65-1 (bottom panel). Data for MT (−) and (+) ends are shown in green and blue respectively. Average rates and population size are indicated. (B). Distribution of MT shortening rates for individual MTs (top panel) or within bundles in the presence of 0.5 µM MAP65-1 (middle panel) and 1.25 µM MAP65-1 (bottom panel). Data for MT (−) and (+) ends are shown in green and blue respectively. Average rates and population size are indicated. Note that in bundles, the density of MTs impaired the reliable detection of (+/−) MT ends depolymerization events; both ends are shown with the same color (middle and bottom panels). In particular, (−) ends were often embedded in complex kymographs, and were underrepresented in the statistical data used in the analysis, as reported shortening rates correspond mainly to MT (+) ends. (C). Duration of MT elongation over time in the absence and in the presence of MAP65-1. (D). Amplitude of MT depolymerization length over time, in the absence or in the presence of MAP65-1. (TIF) [file pone.0056808.s001.tif]

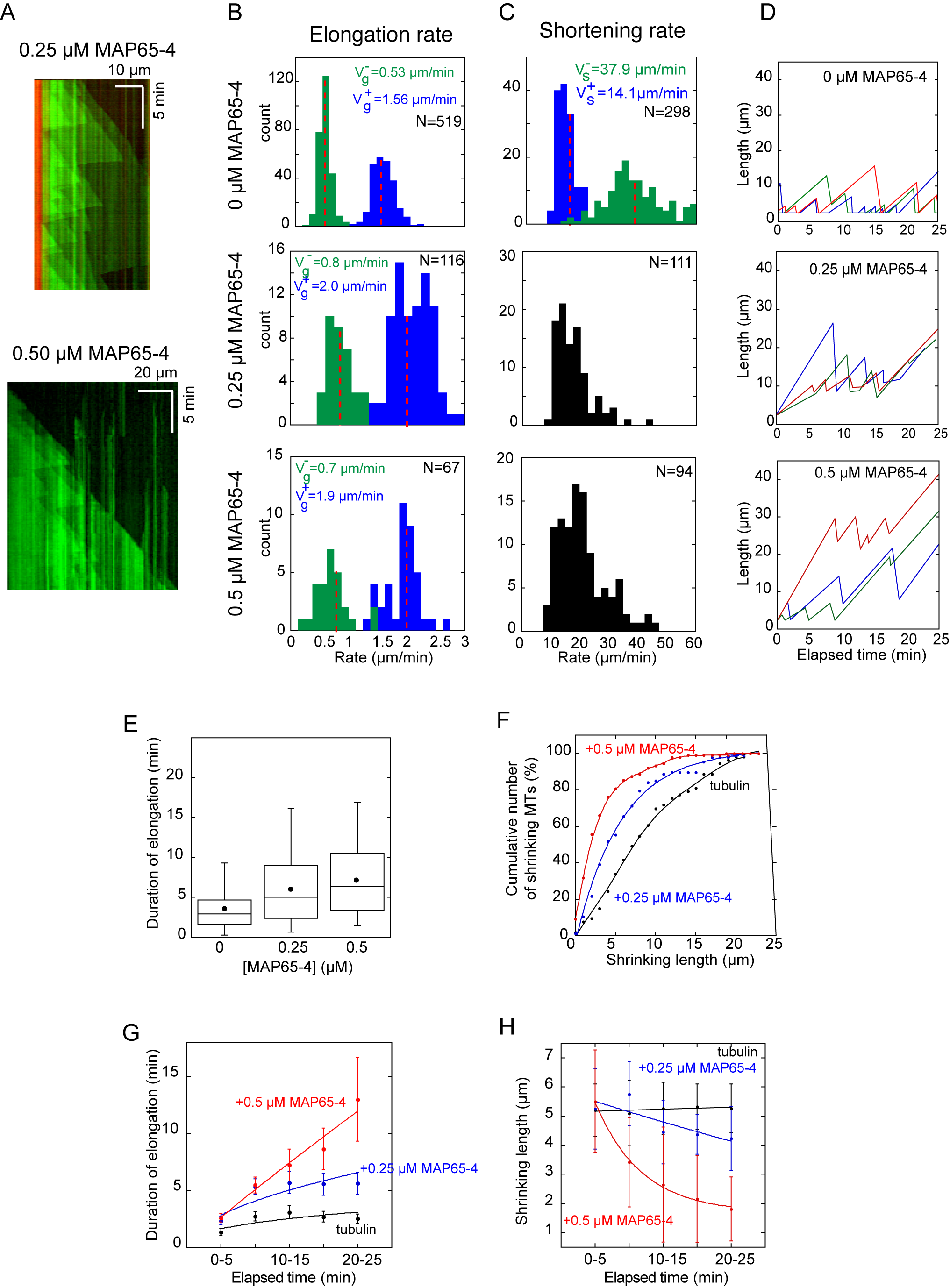

Supplement: Figure S2 — Dynamics of MTs bundled with MAP65-4. (A). Kymographs of MT bundles in the presence of GFP-MAP65-4. (B). Distribution of MT elongation rates for individual MTs (top panel) or within bundles in the presence of 0.25 µM MAP65-4 (middle panel) and 0.5 µM MAP65-4 (bottom panel). Data for MT (−) and (+) ends are shown in green and blue respectively. Average rates and population size are indicated. (C). Distribution of MT shortening rates for individual MTs (top panel) or within bundles in the presence of 0.25 µM MAP65-4 (middle panel) and 0.5 µM MAP65-4 (bottom panel). Average rates and population size are indicated. Data for MT (−) and (+) ends are shown in green and blue respectively. As in Figure S1, we could not reliably distinguish (+) and (−) ends depolymerization events in dense bundles, and both ends are shown with the same color (middle and bottom panels). (D). Length history plot of 3 single MTs in the absence of MAP65-4 (top panel) and 3 MT bundles in the presence of MAP65-4 (middle and bottom panels). (E). Duration of growth phases after a rescue event as a function of MAP65-4 concentration. Dark dots indicate mean values. (F). Depolymerization length of MTs in the presence of MAP65-4. (G). Duration of MT elongation over time in the absence and in the presence of MAP65-4. (H). Variation of MT depolymerization length over time, in the absence or in the presence of M65-4. (TIF) [file pone.0056808.s002.tif]

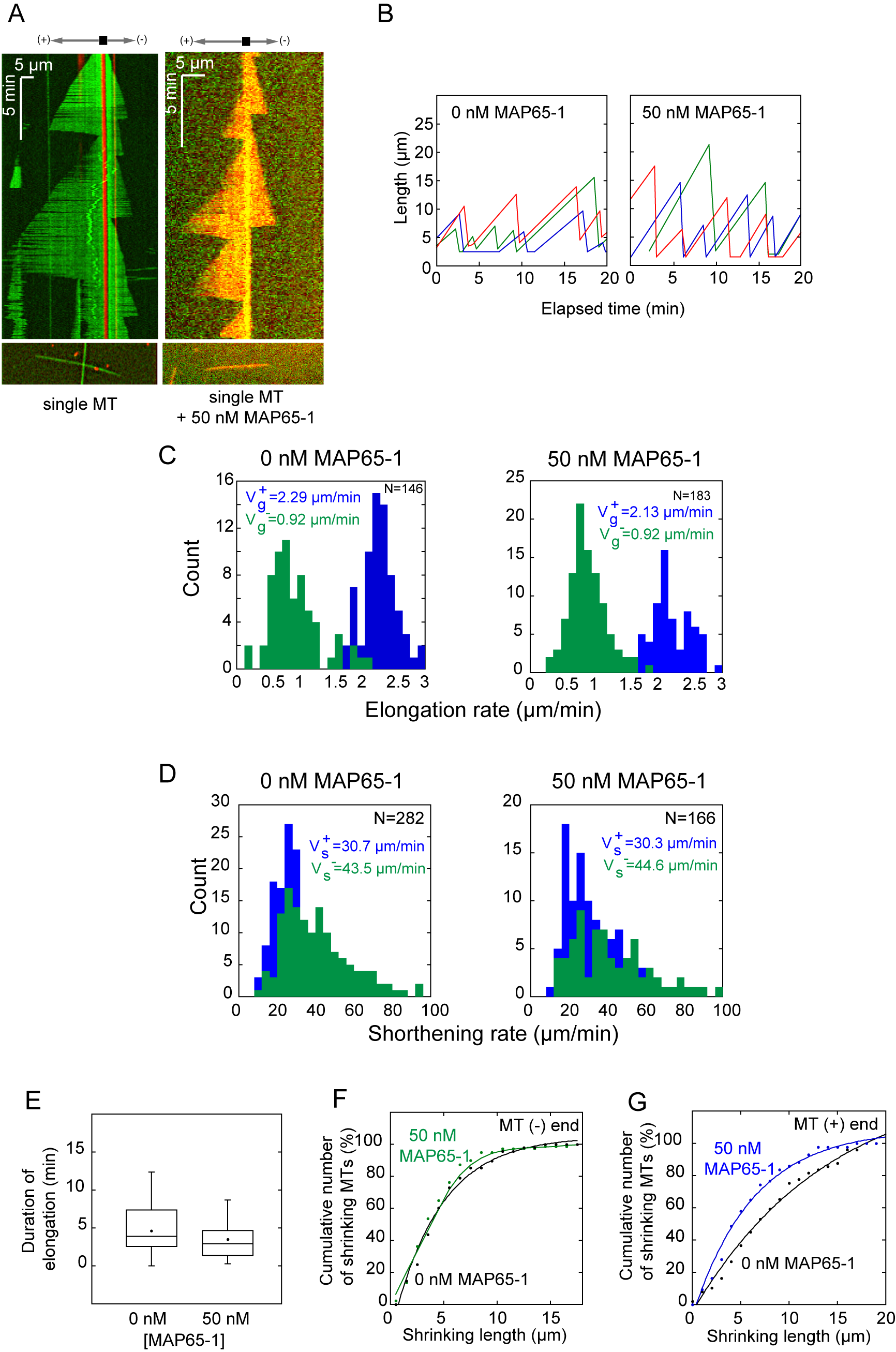

Supplement: Figure S3 — Dynamics of individual MTs in the presence of 50 nM MAP65-1. (A). Kymographs of a single MT that elongates in the absence (left) and in the presence of 50 nM of GFP-MAP65-1 (right). Kymograph is the merge image of Alexa-568 MT (red) elongating from an alexa-568 MT seed (red) in the presence of GFP-MAP65-1 (green). Thus the yellow color reveals the binding of GFP-MAP65-1 on the MT. Schemes on top of the kymographs show the orientation of MT ends. Bottom images show the MT used to draw the kymographs. (B). Length history plot of 3 single MTs in the absence and in the presence of 50 nM MAP65-1. (C–D). Distribution of elongation rates (C) and shortening rates (D) of single MTs in the absence (left column) or in the presence of 50 nM MAP65-1 (right column). Data for MT (−) and (+) ends are shown in blue and green respectively. Average rates and population size are indicated. (E). Duration of elongation of single MTs after a rescue event in the absence and in the presence of 50 nM MAP65-1. Dark dots indicate mean values. (F–G). Depolymerization length of MT minus ends (F) and plus ends (G) in the absence and in the presence of 50 nM MAP65-1. (TIF) [file pone.0056808.s003.tif]

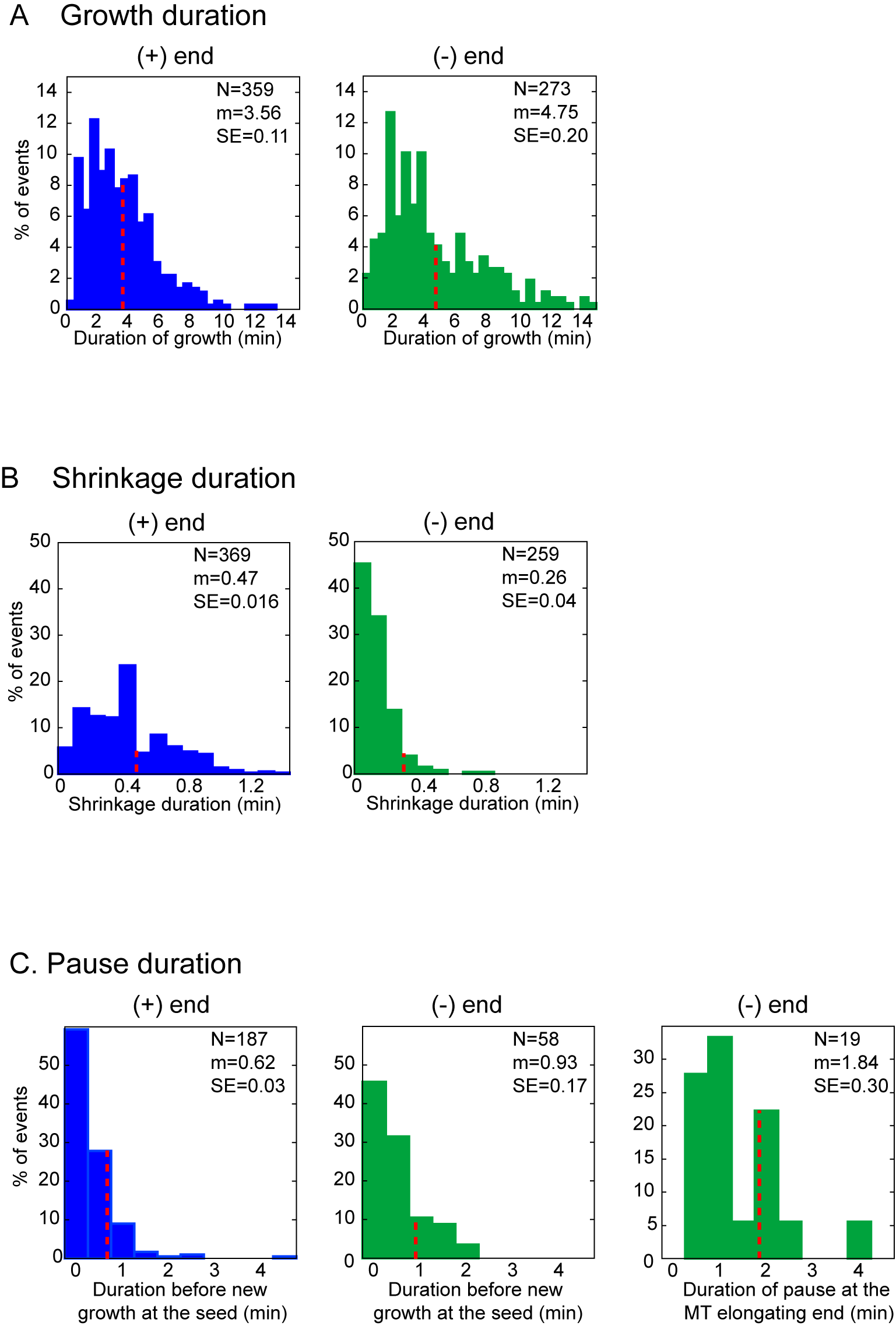

Supplement: Figure S4 — Dynamic parameters of individual MTs in the absence of MAP65. Distribution of the duration of MT elongation (A), the duration of MT shrinkage (B) and the duration of MT pause (C). Data for MT plus and minus ends are shown in blue and green respectively. Population size (N), mean value (m) and standard error (SE) are indicated. (TIF) [file pone.0056808.s004.tif]

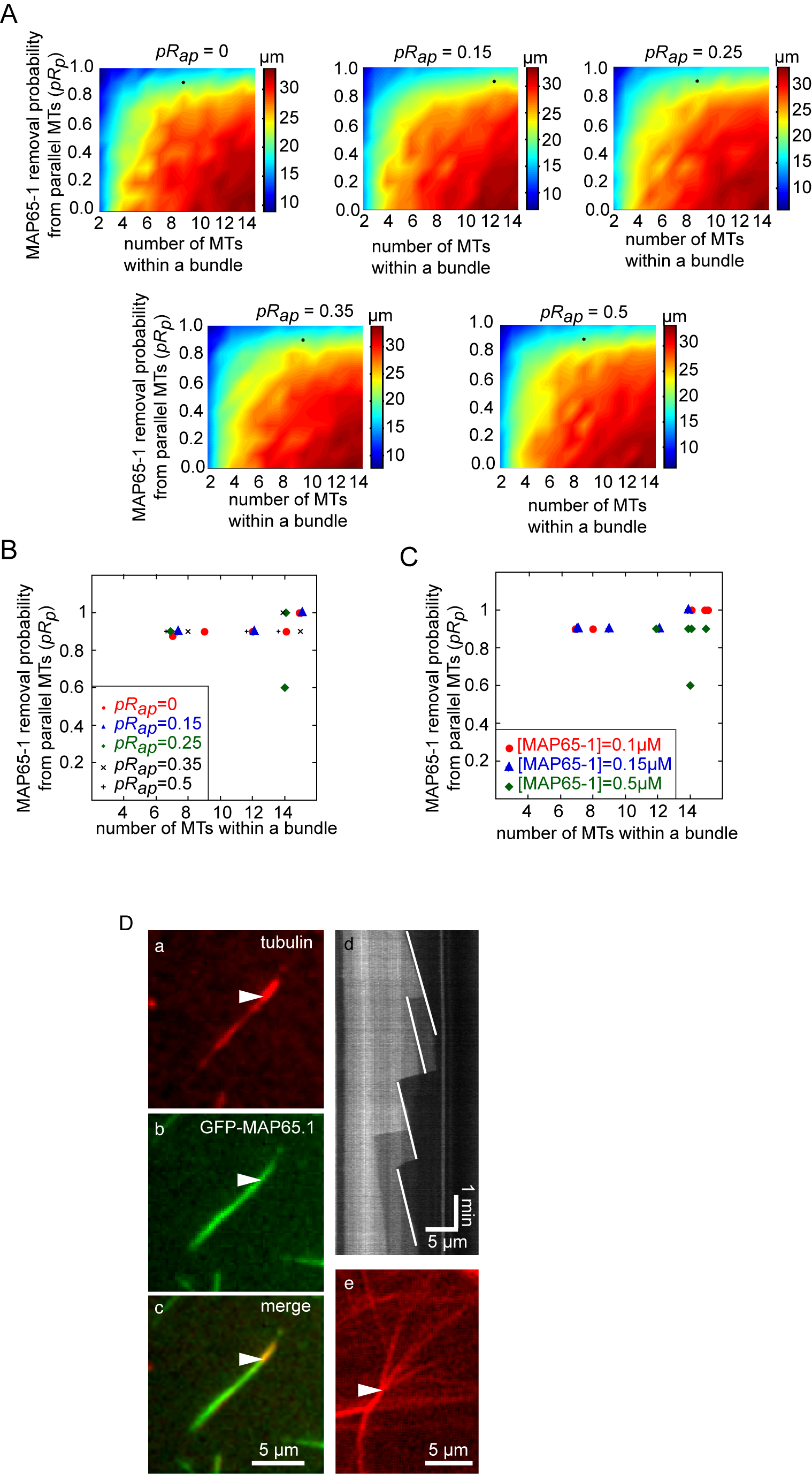

Supplement: Figure S5 — Binding of MAP65-1 to parallel MTs. (A). Examples of calculated MT maximal length at t = 20 min, expressed as a function of pRp (removal probability for MAP65-1 connecting parallel MTs) and the number of MTs. Simulations were repeated for different values of pRap and for a MAP65-1 concentration of 0.5 µM. (B–C). Optimal value for pRp as a function of the number of MTs in the bundle. We determined these values for different removal probability for MAP65-1 connecting anti-parallel MTs (pRap) (B) or various MAP65-1 concentrations (C). Both panels show that pRp is in the range 0.9-1 and is independent of pRap and MAP65-1 concentration. (D). Binding of MAP65-1 (b) to parallel MTs (a) nucleated by axonema (arrowhead). (c) is the merge image of (a) and (b). (d) Kymograph of a MT bundle as in (b). All MT elongation rates are similar (plain lines), showing that MTs are parallel. (e) MTs nucleated from an axonema in the absence of MAP65-1. MTs split apart from the axonema. (TIF) [file pone.0056808.s005.tif]

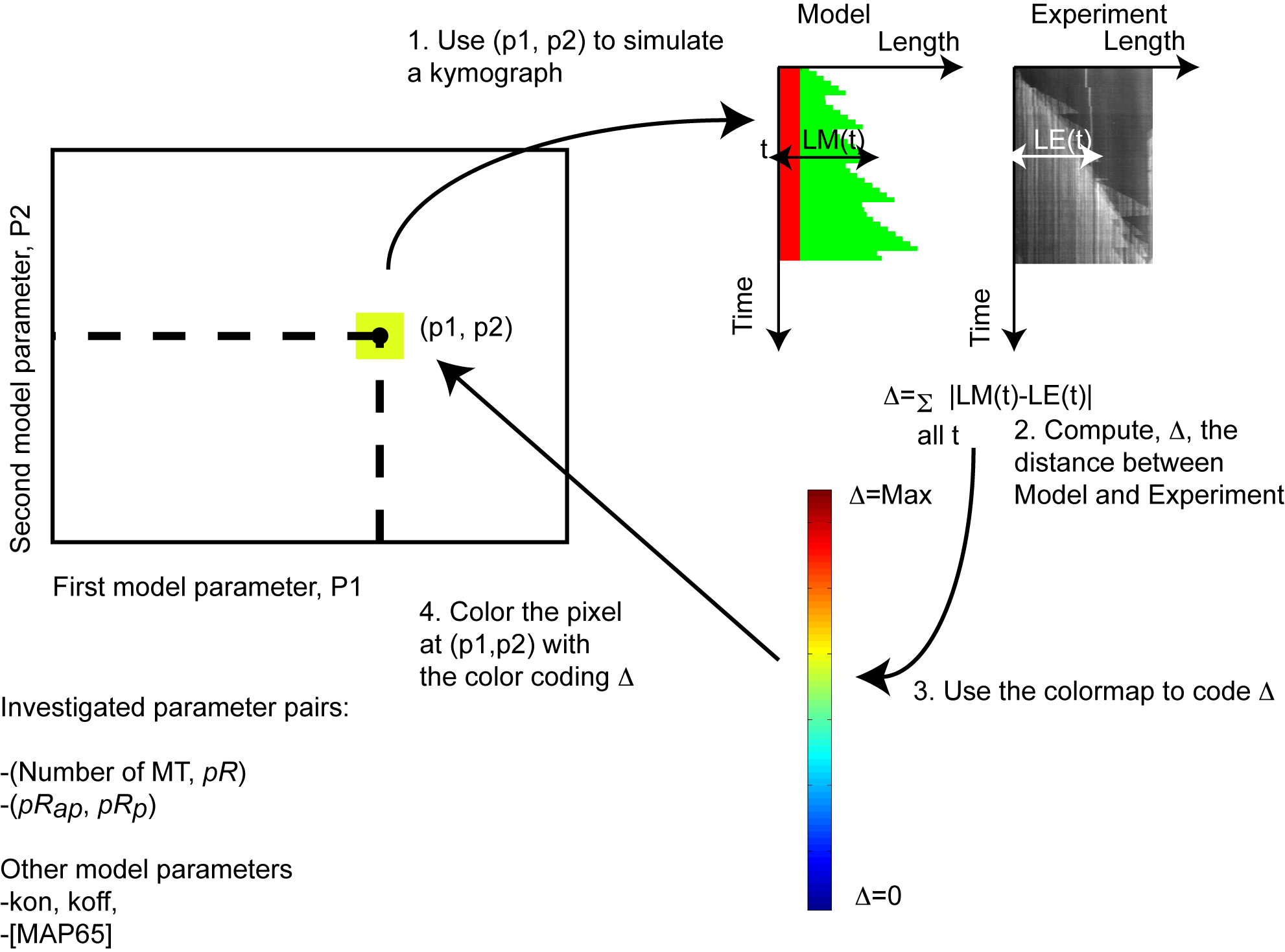

Supplement: Figure S6 — Model simulations and comparison with experiments. The model used in this study depends on kinetic parameters (kon, koff) of MAP65 binding, MAP65 concentrations [MAP65], number of MTs in the bundle and probability factors, pRap and pRp, that govern the MAP65 dynamics during MT depolymerization. Of all these parameters, only (kon, koff) and the MAP65 concentrations are known. We determined the probability factors and the number of MTs that give the best fit with the experiments using the following algorithm: 1. Using a couple of parameters values (p1, p2), we simulated a series of 10 independent kymographs. 2. Then, for each simulated kymograph, we compute, Δ, the distance between the model and the experiment by summing up the difference between the predicted bundle length (LM(t)) and the measured length (LE(t)) for all available time points in both the simulated and experimental kymographs. 3. We code the value of the Δ so that the best match (Δ = 0 or minimum) is dark blue and the worst match is bright red. 4. We color the pixel at position (p1, p2) using the color corresponding to the value of Δ. 5. We repeat the procedure for all pixels in the parameter plane. The parameter combination given the best model-experimental match is directly read out from the position of the dark blue pixel(s) in the parameter plane. (TIF) [file pone.0056808.s006.tif]

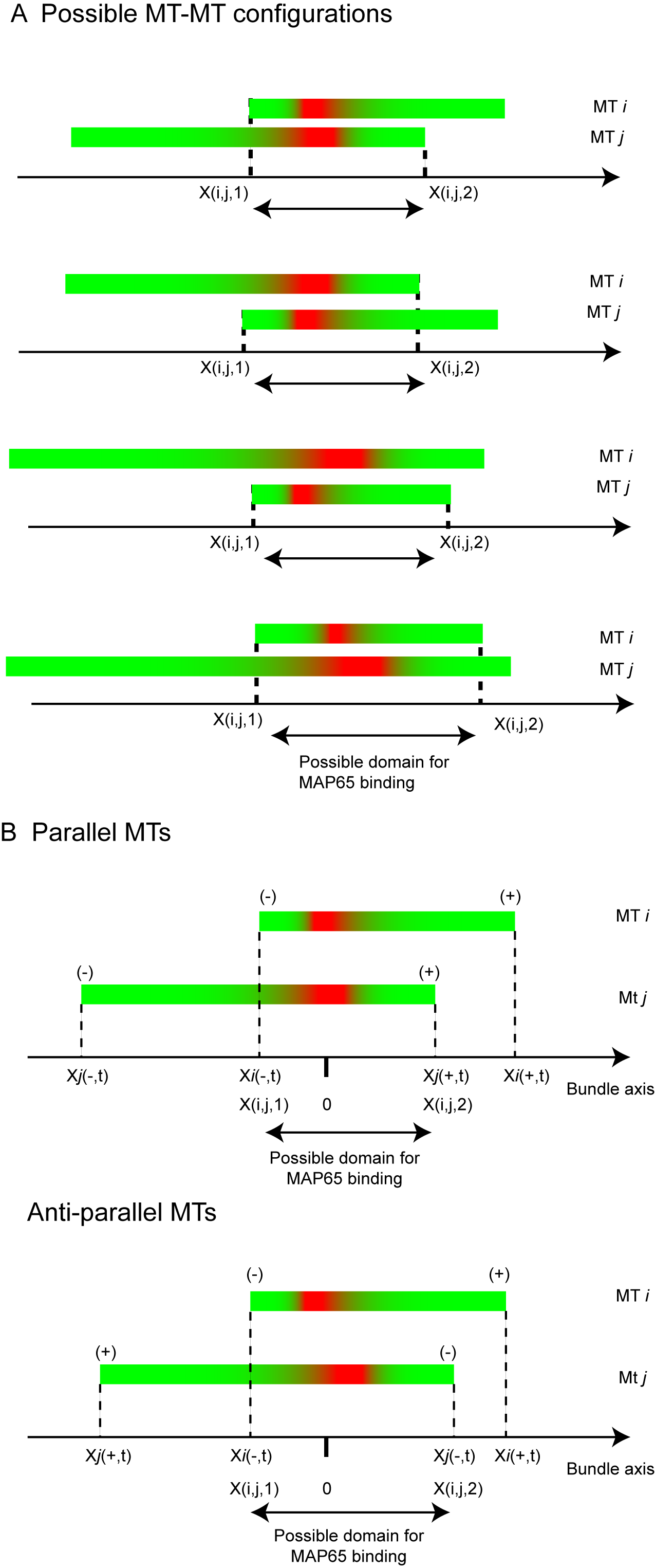

Supplement: Figure S7 — Determination of the spatial domain accessible to MAP65 connections between two microtubules. (A). In the model, we limited our attention to MAP65 connecting MTs in the spatial domain limited by the position X(i,j,1) (left point) and X(i,j,2) (right point). The seed is indicated in red; tubulin in green. (B). Computation of the limit positions X(i,j,1) and X(i,j,2) for parallel (top) or anti-parallel (bottom) MTs using formulae (12–15) in the supplemental text. (TIF) [file pone.0056808.s007.tif]
