# Supplementary material for: MAP65 Coordinate Microtubule Growth during Bundle Formation
Source: PLoS One. 2013 Feb 21;8(2):e56808. doi: 10.1371/journal.pone.0056808 (PMC3578873; doi:10.1371/journal.pone.0056808)
Supplement: Table S1 — List of variables and parameters used in the model for MT dynamics. (DOCX) [file pone.0056808.s008.docx]

**Table S1. List of variables and parameters used in the model for MT dynamics.**

| **Variable** | **Definition** | | **Units** | |  |
| --- | --- | --- | --- | --- | --- |
| *t* | time | s. | |  |  |
| *X(+,t)* | Position of the plus end at time *t* | μm | |  |  |
| *X(-,t)* | Position of the minus end at time *t* | μm | |  |  |
| $T_{+,-}^{G}$ | Growth duration (plus/minus MT ends) | s. | |  |  |
| $T_{+,-}^{S}$ | Shrinkrage duration (plus/minus MT ends) | s. | |  |  |
| $T_{+,-}^{P}$ | Pause duration (plus/minus MT ends) | s. | |  |  |
| $X_{+,-}^{Seed}$ | Position of the plus/minus seed end | μm | |  |  |
| **Parameter** | **Definition** | | **Units** | | **Value** |
| $V_{+}^{G}$ | Growth rate, (+) end | | μm.s^-1^ | | 2.6x10^-2^ |
| $V_{-}^{G}$ | Growth rate, (-) end | | μm.s^-1^ | | 8.8x10^-3^ |
| $V_{+}^{S}$ | Shrinkage rate, (+) end | | μm.s^-1^ | | 2.4x10^-1^ |
| $V_{-}^{S}$ | Shrinkage rate, (-) end | | μm.s^-1^ | | 6.7x10^-1^ |
| $\tau_{+}^{G}$ | Average duration, growth phase, (+) end. | | s. | | 210 |
| $\tau_{+}^{S}$ | Average duration, shrinkage phase, (+) end. | | s. | | 28 |
| $\tau_{+}^{P}$ | Average duration, pause phase, (+) end. | | s. | | 0 |
| $\tau_{-}^{G}$ | Average duration, growth phase, (-) end. | | s. | | 280 |
| $\tau_{-}^{S}$ | Average duration, shrinkage phase, (-) end. | | s. | | 15 |
| $\tau_{-}^{P}$ | Average duration, pause phase, (-) end. | | s. | | 110 |
