# Supplementary material for: MAP65 Coordinate Microtubule Growth during Bundle Formation
Source: PLoS One. 2013 Feb 21;8(2):e56808. doi: 10.1371/journal.pone.0056808 (PMC3578873; doi:10.1371/journal.pone.0056808)
Supplement: Table S2 — Matrix Transition. (DOCX) [file pone.0056808.s009.docx]

**Table S2. Matrix Transition.**

|  | **G** | **S** | **P** | ← After transition |
| --- | --- | --- | --- | --- |
| **G** | **G→G** | **G→S** | **G→P** |  |
| **S** | **S→G** | **S→S** | **S→P** |  |
| **P** | **P→G** | **P→S** | **P→P** |  |
| ↑  Before transition |  |  |  |  |

$P_{-}=\left(\begin{matrix} 0 & 0.95 & 0.05 \\ 0.78 & 0 & 0.22 \\ 1 & 0 & 0 \end{matrix}\right)$, $P_{+}=\left(\begin{matrix} 0 & 1 & 0 \\ 0.45 & 0 & 0.55 \\ 1 & 0 & 0 \end{matrix}\right)$

Letters G, S, P (as for growing ‘G’, shrinking ‘S’ or pausing ‘P’), indicate, respectively, the status of the MT end before (left column) and after (top row) the transition. The intersection of a row and a column gives the probability to make a transition between two MT states as indicated (top row). Left and right matrixes (bottom row) give the values used in simulation for, respectively, the (-) and the (+) end.
