# Supplementary material for: MAP65 Coordinate Microtubule Growth during Bundle Formation
Source: PLoS One. 2013 Feb 21;8(2):e56808. doi: 10.1371/journal.pone.0056808 (PMC3578873; doi:10.1371/journal.pone.0056808)
Supplement: Table S3 — List of parameters used in interactions between MAPs and MTs. (DOCX) [file pone.0056808.s010.docx]

**Table S3. List of parameters used in interactions between MAPs and MTs.**

| **Parameter** | **Definition** | **Unit** | **Value** |
| --- | --- | --- | --- |
| [MAP] | MAP concentration | μM | $0.1\leq\left[MAP\right]\leq1.25$ |
| nMT | Number of MTs in a bundle | - | $2\leq nMT\leq15$ |
| *k_on_* | MAP65-1/4 binding constant, parallel MTs case. | μM^-1^.s^-1^. | 10^-2^ |
| *k_off_* | MAP65-1/4 dissociation constant | s^-1^. | 0 |
|  | Maximal MAP linear density | μm^-1^. | 100 |
| *pR_p_* | Probability to remove a MAP65 connecting parallel MTs during shrinkage. | - | $0\leq{pR}_{p}\leq1$ |
| *pRa_p_* | Probability to remove a MAP65 connecting anti-parallel MTs during shrinkage. | - | $0\leq pR_{ap}\leq1$ |
