# Supplementary material for: MAP65 Coordinate Microtubule Growth during Bundle Formation
Source: PLoS One. 2013 Feb 21;8(2):e56808. doi: 10.1371/journal.pone.0056808 (PMC3578873; doi:10.1371/journal.pone.0056808)
Supplement: Methods S1 — (DOCX) [file pone.0056808.s017.docx]

**SUPPORTING METHODS. Modeling microtubules (MT) growth in the presence of microtubules associated proteins (MAP).**

In section A, we present the kinetic model for the growth and shrinkage phases of isolated MT accounting for growth and shrinkage phases. The kinetic parameters were determined from the analysis of MT dynamics observed with TIRF microscopy in the absence of MAPs. In section B, we introduce the MAP65-1/4 assuming the binding of parallel or anti-parallel MT in a bundle. We hypothesize that the presence of MAP bonds between MT limits MT shortening phases and synchronizes MT growth. In the third section C, we will present how the model is simulated and compared to experimental results.

**A. Kinetics of isolated microtubules**.

We assume that microtubules (MT) grown from taxol-stabilized seeds are aligned along an axis with the initial seed centered at the axis origin.

For a single MT, the positions of its plus (+) and minus (-) ends are given by stochastic, time-dependent variables, respectively, *X(+,t)* and *X(-,t)*. Since the initial orientation of the seed is totally random, the plus (resp. the minus) MT end positions *X(+,t)* and *X(-,t)* have positive/negative abscissae along the horizontal axis with the same probability.

To simplify the model further, we use constant growth/shrinkage rates, assuming that the GTP-tubulin dimers concentration in the medium is largely above the critical concentration of the plus/minus MT ends. During growth or shrinking phases, the position of the MT ends changes depending on the polymerization/depolymerization rates associated with the MT end type (plus/minus) according to

$X(+,t+h)=X(+,t)+hV_{+}^{G}$, (1)

during MT growth and

$X(+,t+h)=X(+,t)+hV_{+}^{S}$, (2)

during MT shrinkage. Note that Eqs. (1) and (2) are valid if a (+) end is located on the right of the origin; for a MT with the opposite orientation, one has

$X(+,t+h)=X(+,t)-hV_{+}^{G}$, (3)

$X\left( +,t+h \right)=X\left( +,t \right)+hV_{\mp}^{S}$. (4)

In equations (1-4), *t* represents the time at which a rescue (resp. catastrophe) event has occurred in the past; in addition, it is implicitly assumed that no catastrophe (resp. rescue) event has occurred in the time interval [*t, t+h*].

Parameters $V_{+}^{G}$ or $V_{+}^{S}$ are, respectively, the growth and shrinkage rates of plus ends experimentally determined from TIRFm analysis (Table S1). The case of minus ends movement is treated using the same formalism:

$X\left( -,t+h \right)=X\left( -,t \right)-hV_{-}^{G}$, (5)

$X\left( -,t+h \right)=X\left( -,t \right)+hV_{-}^{S}$. (6)

for a minus end located on the left of the origin of abscissae. Conversely, a minus end located on the right side of the origin changes its position according to

$X\left( -,t+h \right)=X\left( -,t \right)+hV_{-}^{G}$, (7)

$X\left( -,t+h \right)=X\left( -,t \right)-hV_{-}^{S}$. (8)

The corresponding growth and shrinkage rates for minus ends are listed in Table S2. The presence of non-hydrolysable GTP associated with the tubulin dimers in the MT seed constraints the plus and minus ends positions to be always on the right (resp. left) of the seed ends. For example, if a MT has its plus end on the right side of the origin and if we denote by $X_{+}^{Seed}$(resp. by $X_{-}^{Seed}$) the positions of the plus (resp. minus) seed ends, one has, for all time *t*

$X(-,t)\leq X_{-}^{Seed}<0<X_{+}^{Seed}\leq X(+,t)$.$X\left( -,t \right)\leq X_{-}^{Seed}<0<X_{+}^{Seed}\leq X\left( +,t \right).$ (9)

A similar relation holds for MTs with a reverse orientation

$X(+,t)\leq X_{+}^{Seed}<0<X_{-}^{Seed}\leq X(-,t)$.$X\left( +,t \right)\leq X_{+}^{Seed}<0<X_{-}^{Seed}\leq X\left( -,t \right).$ (10)

In addition to growth and shrinkage phases, we introduced pauses which start when a shrinkage phase was prematurely ended as the position of the MT (plus/minus) end coincides with that of the seed (plus/minus) end, i.e.

$X\left( +,t \right)=X_{+}^{Seed}$ or $X\left( -,t \right)=X_{-}^{Seed}$. (11)

Given (i) the average duration of the different phases determined from the experiments (Table S1), (ii) the rates of growth and shrinkage (Table S1) and (iii) the probabilities for transitions between growth, shrinkage or pause phases (Table S2), one can simulate the position of the plus or minus ends by the following algorithm (here the algorithm is presented for the plus end; the algorithm for the minus end has a similar structure).

We assume that a MT plus end is growing (‘G’), shrinking (‘S’) or pausing (‘P’). The dynamics of MT ends is based on the alternation of G, S or P phases whose duration is a random time drawn from exponential distribution of parameters $\tau_{+}^{G}$, $\tau_{+}^{S}$ or $\tau_{+}^{P}$. These parameters are the average duration of, respectively, growth ($\tau_{+}^{G}$), shrink ($\tau_{+}^{S}$) or pause ($\tau_{+}^{P}$) phases for a (+) end (see Table S1 for the definition and values of these parameters; the average duration of growth, shrink and pause phases of the (-) ends are, respectively, $\tau_{-}^{G}$, $\tau_{-}^{S}$ or $\tau_{-}^{P}$). In addition, we assume that transitions between growth, shrinkage and pause phases are Markovian, i.e. the probability that a particular MT extremity starts a new phase of type $y(t)$ at time *t* depends only on the state $x(t)$ of that extremity just at the transition time. Therefore, a (+/-) MT end is characterized by (i) its phase status ($x(t_{0})\in\left\{G,S,P\right\}\text{ }$), where $t$ is the running time and $t_{0}$ is the beginning of the phase; one has always $t\geq t_{0}$, (ii) the duration of the phase ($T_{+/-}^{x(t_{0})}$). The simulation algorithm consists into two parts: (i) initialization of the MT state at $t=0$ and (ii) MT dynamics simulation.

***Initialization***. At $t=0$, choose the MT end state among (G, S, P) according to the probability distribution ($\frac{\tau_{+}^{G}}{A},\frac{\tau_{+}^{S}}{A},\frac{\tau_{+}^{P}}{A}$)$\left( \frac{\tau_{+}^{G}}{A},\frac{\tau_{+}^{S}}{A},\frac{\tau_{+}^{GP}}{A} \right)$, with $A=\tau_{+}^{G}+\tau_{+}^{S}+\tau_{+}^{P}$. Once the state $x(0)$ is chosen ($x(0)\in\left\{G,S,P\right\}$), draw the duration of this state, denoted by $T_{+}^{x(0)}$, using the exponential distribution of parameter $\tau_{+}^{x\left( 0 \right)}$. Because the initial time is arbitrary, change the duration of the initial phase into $T_{+}^{x\left( 0 \right)}\to{\theta T}_{+}^{x\left( 0 \right)}$, where $\theta$ is a random number drawn from the uniform distribution over the interval [0,1]. In addition, set the velocity of the MT end to $V_{+}^{x(0)}$ (note that $V_{+}^{P}=0$). The case of minus ends is treated using the same initialization procedure. After initialization, a (+/-) MT end is characterized by a set of four parameters ($t_{0},x(t_{0}),T_{+/-}^{x(t_{0})},V_{+/-}^{x(t_{0})}$)$\left( t_{0},x\left( t_{0} \right), T_{+/-}^{x\left( t_{0} \right)},V_{+/-}^{x\left( t_{0} \right)} \right)$, namely the beginning of the phase, the status of the phase, the phase duration and the growth/shrinkage rate.

***MT dynamics***.

1. For all time *t* such that $t_{0}\leq t\leq T_{+}^{x\left( t_{0} \right)}$, use relations (1-2) or (3-4) to update the MT end position using growth/shrinkage rate $V_{+}^{x\left( t_{0} \right)}$. If the MT plus end position coincides with the seed plus end $X_{+}^{Seed}$ at some intermediate time point *t** ($t_{0}\leq t^{*}\leq T_{+}^{x(t_{0})}$), then stop the dynamics and freeze the MT position at $X_{+}^{Seed}$ until completion of the phase.
2. At the end of the $x\left( t_{0} \right)$ phase, i.e. at time $t_{0}+T_{+}^{x\left( t_{0} \right)}$, use the probabilities transitions listed in Table S2 to determine the next MT end state given that the MT was in state $x\left( t_{0} \right)$ at $t_{0}+T_{+}^{x(t_{0})}$Once the new MT end state is chosen (this state is denoted $x\left( t_{0}+T_{+}^{x\left( t_{0} \right)} \right)$, draw its duration from the exponential distribution of parameter $\tau_{+}^{x(t_{0}+T_{+}^{x(t_{0})})}$; change the growth/shrinkage rate to $V_{+}^{x\left( t_{0}+T_{+}^{x\left( t_{0} \right)} \right)}$.
3. Update time, the set of MT parameters according to:

$t_{0}\to t_{0}+T_{+}^{x(t_{0})}$, $x(t_{0})\to x(t_{0}+T_{+}^{x(t_{0})})$, $T_{+}^{x\left( t_{0} \right)}\to T_{+}^{\left( x\left( t_{0} \right)+T_{+}^{x\left( t_{0} \right)} \right)}$, $V_{+}^{x\left( t_{0} \right)}\to V_{+}^{x\left( t_{0}+T_{+}^{x\left( t_{0} \right)} \right)}$.

Then, go back to step 1.

The case of minus ends is treated using the same algorithm.

**B. MAPs bonding and the control of MT kinetics**.

In section A, we presented the algorithm for the dynamics of isolated MT grown from stable seeds. In this section, we consider bundles of MT, with random orientation (i.e. the MT in the bundles can form parallel or anti-parallel MT pairs, with the same probability). The two MAPs under consideration (MAP65-1 and MAP65-4) are known to have different mechanism for MTs bonding and are sensitive to the relative MT orientation in a pair (i.e. parallel or anti-parallel MTs). MAP65-4 is indifferent to the MT pairing whereas MAP65-1 is reported to connect anti-parallel MTs only.

However, the dynamics of MTs bundles in the presence of MAP65-1 is best understood if we relax this constraint and allow bonding between parallel MTs (see the main text for a discussion of this assumption and its consequences for the MT dynamics).

***B1. MAPs binding modeling***.

Assuming that the MTs are regularly arrayed with their positions located on the vertices of a hexagonal lattice (which is equivalent to assume maximal compactness in the bundle), we determine the number of possible pairs between adjacent MTs in the bundle, as shown in Figure S7. Let *i* and *j* be the indexes of two the MTs in a pair. From the positions of the plus/minus ends of MT *i* and *j*, MAPs can connect the two MTs over the spatial domain $[X(i,j,1), X(i,j,2)]$ defined by

$\max\left(X_{j}\left(-,t\right),X_{i}\left(-,t\right)\right)=X\left(i,j,1\right)<0$, (12)

$0<X\left( i,j,2 \right)=min\left( X_{j}\left( +,t \right),X_{i}\left( +,t \right) \right)$, (13)

so that

$X_{i}\left(-,t\right)\leq X\left(i,j,1\right)<X\left(i,j,2\right)\leq X_{i}\left(+,t\right)$ (14)

and

$X_{j}\left(-,t\right)\leq X\left(i,j,1\right)<X\left(i,j,2\right)\leq X_{j}\left(+,t\right)$ (15).

Note that equations (12-15) implicitly assume that the plus (resp. minus) ends have positive (resp. negative) abscissas; otherwise, relations (11-12) should be changed accordingly. The extension of the domain for MAP65 binding and its definition is shown in Figure S7. Therefore, the number of bound MAPs is determined as a binding reaction on a linear lattice with *N_sites_*$N_{sites}$ and controlled by the on/off parameters defined in Table S3. The dynamics of MAP binding on anti-parallel MTs obeys similar equations, using appropriate on/off binding rates and linear MAP density (see Table S3).

The presence of MAPs does not change the kinetic parameters (growth/shrinkage rates; duration of growth and shrinkage phases). Therefore, MTs undergo growth/shrinkage dynamics at their ends as if they were isolated. If the MT dynamics in the pair (*i,j*) is associated with an absolute increase of $X\left( i,j,2 \right)- X(i,j,1)$, the number of available sites for MAPs binding is increased and a wave of bound MAPs follows the growing MT ends. Conversely, if the MT dynamics results in a reduction of the spatial domain $[X(i,j,1), X(i,j,2)]$, one should consider explicitly the interactions between shrinking ends and bound maps. This is the object of the next section.

***B2. Control of MT dynamics by MAPs***.

We assume that MT (*j*) is undergoing shrinkage and that the position of its (+) end, denoted by $X_{j}(+,t)$, is approaching $X\left(i,j,1\right)$; in the meantime, the MT (*i*) is either pausing or elongating. If the position of the (+) end of MT (*j*), $X_{j}\left(+,t\right)$, reaches a MAP position, we assume that the piece of MT (see Figure 5) bound to the MAP and the MAP itself is removed with a probability *pR_p,ap_*${pR}_{p, ap}$ or stays with a probability 1-*pR_p,ap_*${pR}_{p, ap}$ (see Table S2 for the definition of these parameters). If the MAP is removed, shrinkage can resume until a new MAP is encountered; otherwise, the shrinkage phase is stopped prematurely. Note that *pR* does not represent the probability of unbinding the MAP from the MT, an event under the control of parameter k_off_. *pR* represents the possibility that a piece of the MT (one protofilament fragment, Figure 5) can be removed with the MAP itself during depolymerization.

The extent of the MT shrinkage is limited either (1) if the $pR$*pR* is extremely low (close to zero) or (2) if the MAP density is very high. On one hand, low $pR$*pR* favors MAPs resisting MT shrinkage. On the other hand, high MAP density increases the number of MAP removals. Since each of these events has a probability $pR$*pR* to occur, the probability to observe *N* successive removals over a distance *L* is *P(N,L)=( pR_p,ap_)^Lρ^*, where *ρ* $\rho$is the MAP linear density and *N=Lρ*$N=L\rho$. Therefore, if $\rho$*ρ* is large, *P(N,L)* is close to zero.

**C. Model validation**.

We first simulated the dynamics of single MTs that elongate from individual stable seed using the polymerization and depolymerization rates, the frequency of catastrophe and rescue events extracted from kymographs corresponding to the dynamics of individual MTs observed in TIRFm (Table 2; Figure 3A-C; Figure S4; Movie S1). The corresponding simulated kymographs of individual MTs are presented in Figure S5 D-E and Movie S4. Comparison between model and experiments as well the color mapping used in Figure 5 are obtained according the procedure shown in Figure S6.
